# Trust in scientific information mediates associations between conservatism and coronavirus responses in the U.S., but few other nations

**DOI:** 10.1038/s41598-022-07508-6

**Published:** 2022-03-08

**Authors:** Quinnehtukqut McLamore, Stylianos Syropoulos, Bernhard Leidner, Gilad Hirschberger, Kevin Young, Rizqy Amelia Zein, Anna Baumert, Michal Bilewicz, Arda Bilgen, Maarten J. van Bezouw, Armand Chatard, Peggy Chekroun, Juana Chinchilla, Hoon-Seok Choi, Hyun Euh, Angel Gomez, Peter Kardos, Ying Hooi Khoo, Mengyao Li, Jean-Baptiste Légal, Steve Loughnan, Silvia Mari, Roseann Tan-Mansukhani, Orla Muldoon, Masi Noor, Maria Paola Paladino, Nebojša Petrović, Hema Preya Selvanathan, Özden Melis Uluğ, Michael J. Wohl, Wai Lan Victoria Yeung, B. Burrows

**Affiliations:** 1 Psychological & Brain Sciences, University of Massachusetts Amherst, Tobin Hall, 135 Hicks Way, Amherst, MA 01003 USA; 2 IDC, Herzliya, Israel; 3 Universitas Airlangga, Surabaya, Indonesia; 4 Max Planck Institute for Research On Collective Goods, Bonn, Germany; 5 University of Warsaw, Warsaw, Poland; 6 Universiteit van Amsterdam, Amsterdam, The Netherlands; 7 Université de Poitiers, Poitiers, France; 8 University of Paris Nanterre, Nanterre, France; 9 Sungkyunkwan University, Seoul, Republic of Korea; 10 Universidad Nacional de Educacion a Distancia, Madrid, Spain; 11 Bloomfield College, Bloomfield, USA; 12 University of Malaya, Kuala Lumpur, Malaysia; 13 University of Edinburgh, Edinburgh, UK; 14 University of Milano-Bicocca, Milan, Italy; 15 De La Salle University, Manila, Philippines; 16 University of Limerick, Limerick, Republic of Ireland; 17 Keele University, Keele, UK; 18 University of Belgrade, Belgrade, Serbia; 19 University of Queensland, Brisbane, Australia; 20 University of Sussex, Brighton, UK; 21 Carleton University, Ottawa, Canada; 22 Lingnan University Hong Kong, Tuen Mun, China; 23 University of Illinois at Urbana-Champaign, Champaign, USA; 24 University of Trento, Trento, Italy; 25 University of Wuppertal, Wuppertal, Germany

**Keywords:** Psychology, Human behaviour

## Abstract

U.S.-based research suggests conservatism is linked with less concern about contracting coronavirus and less preventative behaviors to avoid infection. Here, we investigate whether these tendencies are partly attributable to distrust in scientific information, and evaluate whether they generalize outside the U.S., using public data and recruited representative samples across three studies (*N*_total_ = 34,710). In Studies 1 and 2, we examine these relationships in the U.S., yielding converging evidence for a sequential indirect effect of conservatism on compliance through scientific (dis)trust and infection concern. In Study 3, we compare these relationships across 19 distinct countries. Although the relationships between trust in scientific information about the coronavirus, concern about coronavirus infection, and compliance are consistent cross-nationally, the relationships between conservatism and trust in scientific information are not. These relationships are strongest in North America. Consequently, the indirect effects observed in Studies 1–2 only replicate in North America (the U.S. and Canada) and in Indonesia. Study 3 also found parallel direct and indirect effects on support for lockdown restrictions. These associations suggest not only that relationships between conservatism and compliance are not universal, but localized to particular countries where conservatism is more strongly related to trust in scientific information about the coronavirus pandemic.

While the coronavirus pandemic has affected hundreds of millions of people, the impact has not been evenly distributed. The United States, despite comprising approximately 4.25% of the global population, has led the world in both coronavirus cases and deaths throughout 2020 and 2021^1^. While there are societal-level reasons why the pandemic has hit the U.S. particularly hard (e.g., delayed pandemic responses, mismanagement by leadership; see^2^, individual attitudes and behaviors have helped shape how communities are affected by the pandemic. Over the course of the pandemic, social scientists have striven to understand factors that underlie such individual differences^3^. Among Americans, empirical evidence suggests that one such factor is political ideology. A number of recent studies suggest that, in American samples, conservatism predicts less concern about contracting the coronavirus and less engagement in behaviors (i.e., social distancing) that prevent its spread^4–7^.

From one perspective^8^, these patterns are puzzling because political conservatives are thought to be more vigilant against physical threats (such as infectious disease) than liberals^9–11^. From another, these patterns are consistent with recent evidence that conservatives and liberals may instead be sensitive to different specific types of collective threats based upon their political identities^12–14^. Among Americans, these patterns are consistent with existing literature linking conservatism with lower trust in scientific information^15^. Emerging evidence over the course of the coronavirus pandemic suggests that trust in science is a critical factor underlying threat perception and compliance with preventative measures in the coronavirus pandemic^16–18^. These emergent findings suggest that conservatism has direct and indirect associations with compliance with preventative measures through trust in science and risk perceptions. Here, we investigated whether, among Americans, conservatism is indirectly associated with concern about contracting the coronavirus (i.e., personal threat perceptions) and preventative behaviors (e.g., social distancing) through (dis)trust in scientific information about the coronavirus pandemic, using larger, more representative samples than prior work. We further investigated whether these patterns generalized beyond the United States, a context in which scientific trust in general, and about the coronavirus pandemic in particular, is highly polarized [see^15^], using a large cross-national dataset.

## Trust in scientific and medical information

One of the strongest predictors of threat perceptions from coronavirus infection, compliance with preventative behaviors, and support for lockdown restrictions, is how much participants trust information from scientists and scientific institutions^16,18^. Existing evidence gleaned from convenience samples during the early months of the coronavirus pandemic suggests that conservatism may be an important antecedent of trust in scientific information within the context of the coronavirus pandemic^16,18,19^. This tendency is consistent with other findings that components of conservatism (e.g., resistance to change, religiosity, and traditionalism) can lead conservatives to distrust scientific findings^20,21^. However, this distrust may be localized to particular scientific areas (e.g., climate change)^22,23^, and there is also evidence that liberals may also be skeptical of ideologically inconsistent information^24–26^. Thus, associations between conservatism and specific distrust of scientific and medical information about the coronavirus may be less due to inherent features of conservatism and more due to politicization of the pandemic.

Where such politicization of scientific areas is severe, such as the United States^15,22,24,27^, these implications can be particularly deleterious. American conservatives are distrustful of scientists and scientific information^24–26^. Yet, associations between conservatism and (dis)trust in scientific information specific to the coronavirus pandemic may not completely generalize outside the United States, and evidence from convenience samples suggest that the correlation is stronger in the United States than in other countries^17,19^. For comparison, the case of climate change skepticism may be instructive. Among Americans, distrust in scientific information is promoted in conservative media networks^27–29^, and conservatism is among the strongest predictors of skepticism in anthropogenic climate change^30–32^.

Yet, meta-analytic cross-national data reveals that this association is far stronger in the U.S. than anywhere else measured^33^. Indeed, Hornsey and colleagues (2018) point out that three-quarters of the countries they surveyed displayed no significant meta-analytic relationship between conservatism and climate change skepticism. One possible explanation for this cross-national variation was that in countries where such a relationship was found, conservative politicians and media frame “green” goals as incompatible with their ideology and spread misinformation about climate change^33,34^. By analogy, associations between conservatism and the coronavirus pandemic may emerge most strongly in countries where the coronavirus pandemic has been particularly politicized [see also^14,15,29^].

## Political ideology & threat perception during the coronavirus pandemic

Theoretically, heightened threat sensitivity has been viewed as an antecedent of right-wing political ideologies^8,35–37^. Evidence suggests that political conservatives have higher needs for stability and security, motivating sensitivity and responsivity toward potential threats^8,17,37,38^. However, this evidence also suggests that conservatism is linked more specifically to sensitivity to proximal, immediate physical threats^14,38^, whereas there are circumstances in which liberals and leftists are more sensitive than conservatives to more global, abstract (but still physical) threats such as climate change or health care infrastructure^38,39^. The nature of the threat the coronavirus pandemic represents could therefore lead conservatives to be less concerned about the pandemic.

However, Plohl and Musil (2020) argue that perceived risk associated with the pandemic should be predicted by the extent to which participants trust scientific information because scientific and medical authorities are the primary source of information about the threat. If, as their study and others suggest, conservatism serves as an antecedent of trust in science (see^16–19^), then conservatism may be associated with risk perceptions through the mechanism of (dis)trust in scientific information. However, this association could only be expected to emerge when conservatism is associated with trust in scientific information. In general, such a link can be observed in the U.S. across multiple contexts^24,40–43^. More specifically, conservatives in the U.S. may express less concern about contracting the virus and engage in less protective behaviors to avoid spreading it (4) in part because conservative politicians and media within that country explicitly downplay the risk (^7,44–47^; see also^48^). Notably, trust in then-President Donald Trump was among the strongest predictors of coronavirus responses^49^.

In contrast, such effects were not observed among Germans, for whom conservatism was positively associated with concern about contracting the coronavirus^50^, as predicted by prior theory^8^. Similar effects were observed in Israel, whose then-Prime Minister Netanyahu instead emphasized the threat of the pandemic^51^, taking a hard line with strict lockdowns that went so far as to outlaw mass protests altogether during the pandemic, yielding opposition from more left-wing citizens of Israel^52^. These findings further suggest that the patterns observed by Plohl and Musil (2020) may not generalize globally.

## Research overview

Plohl and Musil (2020), Pagliaro and colleagues (2021), and Sulik and colleagues (in press) have described indirect associations between conservatism and compliance with preventative measures during the coronavirus pandemic. However, these studies relied upon convenience samples, and did not test whether these indirect effects were stronger in the U.S. compared to in other countries. In three studies (*N*_total_ = 34,710), we tested for associations between political ideology with attitudes and beliefs towards the coronavirus pandemic through trust in scientific information about the coronavirus pandemic and concern about contracting the coronavirus using representative, high-powered samples from multiple research teams. We hypothesized that among Americans, conservatism would indirectly relate to concerns about contracting the coronavirus through trust in scientific authorities and institutions (H1), but that these effects should be strongest where reactions to coronavirus are strongly polarized, particularly the United States (H2).

The data presented in Studies 1a–1b utilize public, representative samples from the ANES 2020 Social Media Study before and after the 2020 Presidential election. In Studies 2a–2b, we present parallel data from two distinct representative samples of Americans during the same time period, allowing for replication of effects between different research teams as well as across time periods. In Study 3, we analyzed data from a previously collected (May–July 2020) large, three-wave, cross-sectional study of attitudes and behaviors in the coronavirus pandemic from 21 countries to evaluate how present such relationships are across the world.

## Studies 1a–1b

We made use of publicly available data from large, representative samples of the American population from the American National Election Studies (ANES). Specifically, data for Studies 1a–1b were obtained from the ANES Social Media Study, conducted between before (Study 1a) and after (Study 1b) the 2020 U.S. presidential election. All data and materials for this study can be found here: https://electionstudies.org/data-center/2020-social-media-study/. With these data, we again examined associations between conservatism, concern about contracting the coronavirus, and trust in science and scientific information. Owing to the vastly different set of variables available in this data, we examined trust in a specific organization dispensing scientific and medical information in Studies 1a–1b, specifically, the Center for Disease Control (CDC). While informed consent for use of this data as publicly available data was obtained, the researchers never directly interacted with these participants, as Studies 1a–1b conduct analyses of public data. The analyses in these studies were not pre-registered.

## Methods

### Participants

A sample of 5750 Americans was collected by ANES in T1 (August 2020). Here, 2905 participants were male (50.52%) and 2845 were female (49.48%). At T1, 3983 participants identified as White (69.27%), 611 as Black or African American (10.63%), 736 as Hispanic (7.30%), and 420 as other racial or ethnic identities, including Asian Americans (7.30%). The average age was 49.51 years (*SD* = 16.27).

For T2 (November 2020) 5277 participants took part in the survey. In this sample, 2664 participants were male and 2613 were female. In terms of race and ethnicity, 3702 participants identified as White, 544 as Black, 647 as Hispanic, and 384 as Asian, mixed, or some other race/ethnicity. The average age was 50.91 years (*SD* = 16.78). Further demographic information for both studies is presented in Table S1.

### Materials and procedure

From the ANES Social Media Study, the variables relevant to our investigation were a measure of political ideology, concern about contracting the coronavirus personally, and confidence in the CDC (serving as a measure of trust in a specific source of scientific information). Political ideology was measured using a single 7-point Likert scale (1 = Very Liberal; 7 = Very Conservative; midpoint = 4, “Neither liberal nor Conservative”), pre-election: *M* = 4.07, *SD* = 1.78; post-election: *M* = 4.04, *SD* = 1.81. Concern about contracting the virus was also measured using a single item (“How worried are you personally about getting the coronavirus (COVID-19)?”; 1 = Not at all worried; 5 = Extremely worried), pre-election: *M* = 2.72, *SD* = 1.21; post-election: *M* = 2.78, *SD* = 1.21. One item, (“How much confidence do you have in the U.S. Centers for Disease Control (CDC)?”), measured on a 1 (“None”) to 5 (“A great deal”) Likert scale was used as a proxy for trust in scientific information sources, *M* = 3.15, *SD* = 1.12; post-election: *M* = 3.34, *SD* = 1.16.

### Data analysis

All data analysis in Studies 1a–1b was conducted using SAS 9.4^53^. As the data collected in Studies 1a–1b were non-probability samples, with the aim of matching the population of the United States, ANES recommends the use of sampling weights for analyses with these data (see https://electionstudies.org/data-center/2020-social-media-study/). Thus, all analytical procedures accounted for the sampling weights provided by ANES. For preliminary analyses, we used the *proc corr* command (accounting for sample weights) to probe (weighted) correlations between conservatism, confidence in the CDC, and concern about contracting the coronavirus. For our main analyses probing indirect effects, the *proc calis* command (accounting for sample weights) was used to construct a path model using maximum likelihood estimates.

## Results

### Correlations

In the T1 sample, conservatism was negatively correlated with confidence in the CDC, *r*(5733) = − 0.253, *p* < 0.001, and with concern about contracting the coronavirus, *r*(5737) = − 0.336, *p* < 0.001. Confidence in the CDC was positively correlated with concern, *r*(5741) = 0.223, *p* < 0.001.

Similarly, in the T2 sample, conservatism was negatively correlated with confidence in the CDC, *r*(5261) = − 0.367, *p* < 0.001, and with concern about contracting the coronavirus, *r*(5263) = − 0.330, *p* < 0.001. Confidence in the CDC was positively correlated with concern, *r*(5272) = 0.277, *p* < 0.001.

### Indirect effect test

For both the pre and post-election surveys, we conducted tests for indirect effects of conservatism on concern through a proxy for trust in scientific institutions, here confidence in the CDC, controlling for age, binarized gender (male = 1, female = − 1), income, and education level.

**Figure 1:**
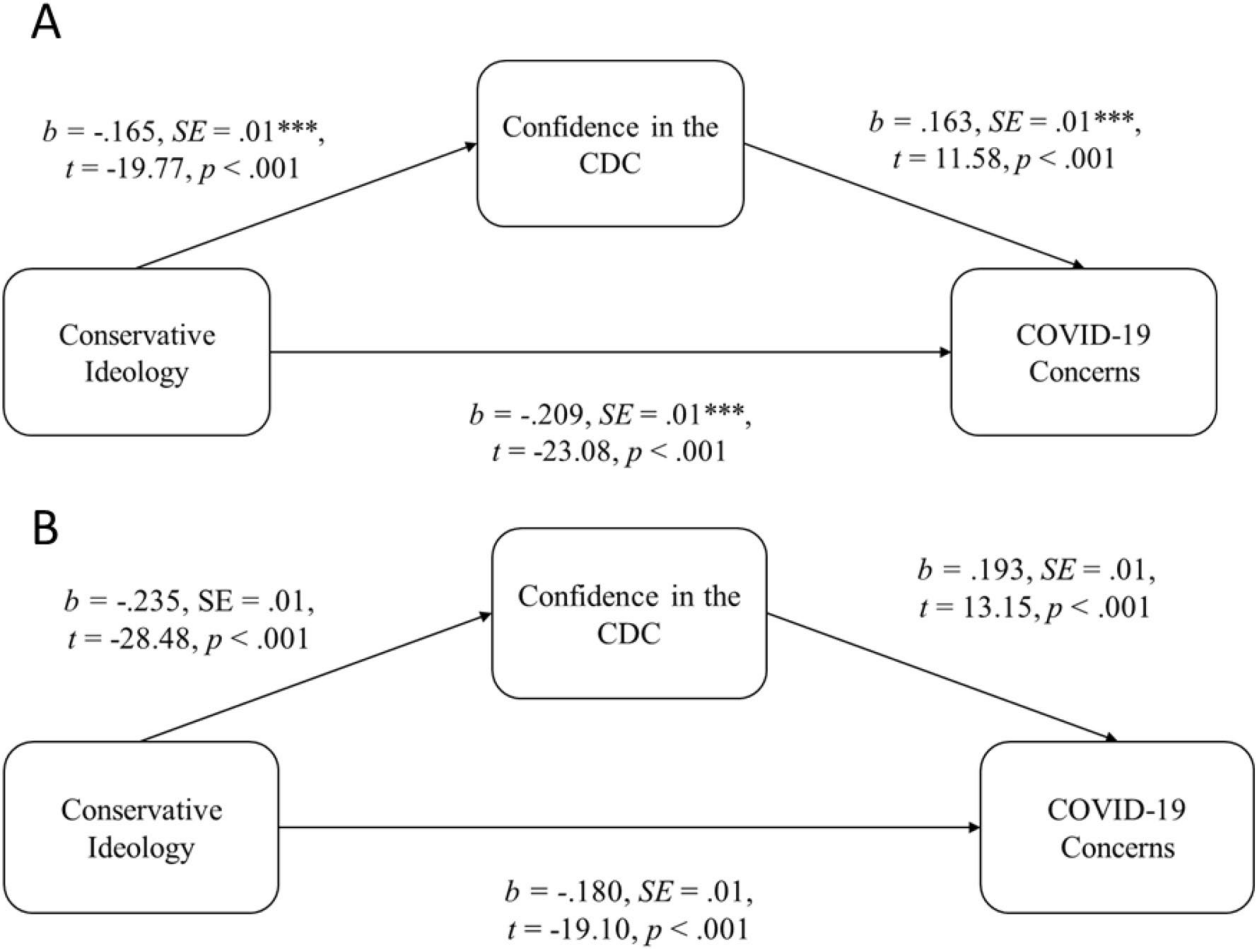
(**A**, **B**) Indirect effect test (*proc calis*, SAS 9.4, Path Model with weighted coefficients, Maximum Likelihood testing method) of conservative ideology on concerns about COVID-19 via the mechanism of trust in science, controlling for binarized gender (male = 1, female = − 1), education level, income level, and age, in Study 1a (**A**, top) and Study 1b (**B**, bottom). Figure was constructed in Microsoft PowerPoint with manually input data copied from SAS 9.4 output. *Note:* ***p < .001.

In Study 1a, we found a significant indirect effect whereby conservatism was associated with less confidence in the CDC, which was associated with more concern about contracting the virus, thus conservatism indirectly was linked to less concern through confidence in the CDC, *b* = − 0.027, *SE* = 0.003, *t* = − 9.99, *p* < 0.001 (Fig. 1A). Yet, the direct effect of conservatism on concern remained significant, *b* = − 0.209, *SE* = 0.009, *t* = − 23.08, *p* < 0.001; total effect: *b* = − 0.236, *SE* = 0.009, *t* = − 26.63, *p* < 0.001. The indirect effect observed represented 11.39% of the total effect.

In Study 1b, we also found a significant indirect effect, such that conservatism was associated with less confidence in the CDC, which was associated with more concern about contracting the virus, thus conservatism indirectly related to less concern through confidence in the CDC, *b* = − 0.045, *SE* = 0.004, *t* = − 11.94, *p* < 0.001 (Fig. 1B). Once again, the direct effect of conservatism on concern remained significant, *b* = − 0.180, *SE* = 0.009, *t* = − 19.10, *p* < 0.001; total effect: *b* = − 0.226, *SE* = 0.009, *t* = − 25.28, *p* < 0.001. The indirect effect observed represented 20.12% of the total effect. The estimated models for both the pre-election survey (*χ*^*2*^(8) = 304.70, *p* < 0.001, CFI = 0.90, RMSEA = 0.08, SRMR = 0.04) and the post-election survey (*χ*^*2*^(10) = 313.27, *p* < 0.001, CFI = 0.97, RMSEA = 0.08, SRMR = 0.04) displayed good fit under Kline (2016) parameters.

## Studies 2a–2b

We collected data in two nationally representative samples, before and after the 2020 U.S. presidential election. Given our ability to control the survey instruments, we conceptually replicated our findings with more comprehensive and detailed measures of trust in science and concern for COVID-19. Further, we also expanded on our findings by investigating the additional outcome of compliance to COVID-19 recommendations. Informed consent was obtained by the researchers for all participant data used in Studies 2a–2b.

## Methods

### Participants

While these analyses were not pre-registered, the sampling method was pre-determined for both samples (see AsPredicted #48042 and #53678). All research in Studies 2a–2b received approval from the University of Massachusetts Amherst Institutional Review Board (IRB Protocol #2317). All activities involving these participants were approved by the University of Massachusetts Amherst IRB and complied with all APA guidelines and policies for human subjects research.

#### Study 2a

The first sample (*N* = 1672 Americans) was collected via CloudResearch^54^ in late September 2020. CloudResearch includes features which allow the recruitment of census-matched samples (see https://www.cloudresearch.com/resources/blog/how-to-gather-demographically-representative-samples-in-online-studies/). In this sample, 595 (43.98%) participants were male, 753 (55.65%) were female, and 5 were nonbinary or other genders (0.37%), while 319 provided no response for gender identity. In terms of participant race and ethnicity, 957 (70.78%) identified as White, 158 (11.69%) as Black, 142 (10.50%) as Hispanic or Latino, 58 -(4.29%) as Asian, 15 (1.11%) as Native American, and 22 (1.63%) as other races/ethnicities, while 320 participants provided no response. The average age was 43.80 years (*SD* = 17.36).

#### Study 2b

The second sample (*N* = 1431 Americans) was collected via Lucid in early December 2020. Lucid is another crowdsourcing website capable of recruiting a demographically diverse and nationally representative sample at a low cost with good reliability^54^. In this sample, 555 (47.93%) participants were male, 597 (51.55%) were female, 6 (0.52%) were non-binary or other genders, and 273 provided no response for gender. In terms of race and ethnicity, 840 (72.66%) participants identified as White, 133 (11.51%) as Black, 87 (7.53%) as Hispanic or Latino, 52 (4.50%) as Asian, 19 (1.64%) as Native American, 25 (2.16%) as other races/ethnicities, and 275 provided no response. The average age was 45.46 years (*SD* = 16.80).

### Materials and procedure

Participants were presented with a battery of measures followed by demographic questions. They were then subsequently debriefed about the purpose of the study. For the purposes of the current investigation, were solely interested in measures of ideology, trust in science, concern about the pandemic, and compliance intentions. The same measures that follow were displayed in the same manner and scale across both studies.

#### Conservative ideology

We measured individual’s political ideology with a single-item measure obtained from the most recent YouGov poll (“In general, I am…”). Scores ranged from “1 = Very liberal” to “7 = Very Conservative”, (Study 2a: *M* = 3.65, *SD* = 1.76; Study 2b: *M* = 3.86, *SD* = 1.85).

#### Trust in science

Given that both trust in science in general, as well as trust in specific scientific institutes was found to be an antecedent of concerns for the coronavirus pandemic, we incorporated both components in our measure of trust in science. These items were preceded by the stem “To what extent do you trust information about the coronavirus if it comes from each of the following information sources?” and were measured on a 1–5 Likert scale (1 = not at all, 5 = very much). These were: (1) The Center for Disease Control and Prevention (CDC); (2) Scientific authorities and professionals (e.g., epidemiologists, virologists); and (3) Medical professionals (e.g., doctors, nurses, surgeons, EMTs). This composite demonstrated good reliability in both Study 2a (*M* = 3.87, *SD* = 0.95, *a* = 0.82), and Study 2b (*M* = 3.91, *SD* = 0.96, *a* = 0.83).

#### Coronavirus concern

A single item was used to measure concern about contracting the coronavirus, “Which, if any, of the following statements describes your feelings toward getting the coronavirus?” measured on a 1 to 4 point Likert scale (I am not at all/not very/ somewhat/ very scared I will contact the coronavirus (COVID-19), in both Study 2a, *M* = 2.73, *SD* = 0.99, and Study 2b, *M* = 2.91, *SD* = 0.97.

#### Coronavirus compliance

Four items on 0–10 Likert scale ranging from “0 = Never” to “10 = All the Time”, were used to capture individual differences in reported compliance to COVID-19 recommendations (e.g., “How often have you avoided social gatherings due to COVID-19?”, “How often have you avoided non-essential travel?”). This composite demonstrated good reliability in both Study 2a (*M* = 7.89, *SD* = 2.31, *a* = 0.87), and Study 2b (*M* = 8.17, *SD* = 2.23, *a* = 0.87).

### Data analysis

All data analysis for Studies 2a–2b was conducted using SAS 9.4^53^. Unlike Studies 1a–1b, the samples we collected here were not recruited using non-probability methods, and thus weighting was not necessary. Similar to Studies 1a–1b, however, preliminary analyses involved computing zero-order correlations between conservatism, trust in science, concern about contracting the coronavirus, and coronavirus compliance using the *proc corr* command. Sequential indirect effects were modeled using Hayes’s PROCESS macro, version 3, for SAS 9.4^55^ with 10,000 bootstrap samples, as this was possible given the non-necessity of weighting samples in these studies (see https://processmacro.org/faq.html).

## Results

### Correlations

In both studies, having a more conservative political ideology was associated with less trust in science, less concerns about COVID-19, and less compliance to COVID-19 recommendations. Trust in science was positively associated with more COVID-19 concerns and compliance. Finally, COVID-19 concerns were positively associated with compliance (Table 1).

**Table 1:** Bivariate correlations for Study 2a (below the diagonal) and Study 2b (above the diagonal).

	1	2	3	4
1. Conservative Ideology	–	− 16	− 15	− 0.08
2. Trust in Science	− 0.16	–	0.31	0.42
3. COVID-19 Concerns	− 0.22	0.27	–	0.41
4. COVID-19 Compliance	− 0.21	0.49	0.41	–

All coefficients are higher than .10 are significant at *p* < 0.001, all coefficients below .10 are significant at *p* < .01.

### Indirect effect test

**Figure 2:**
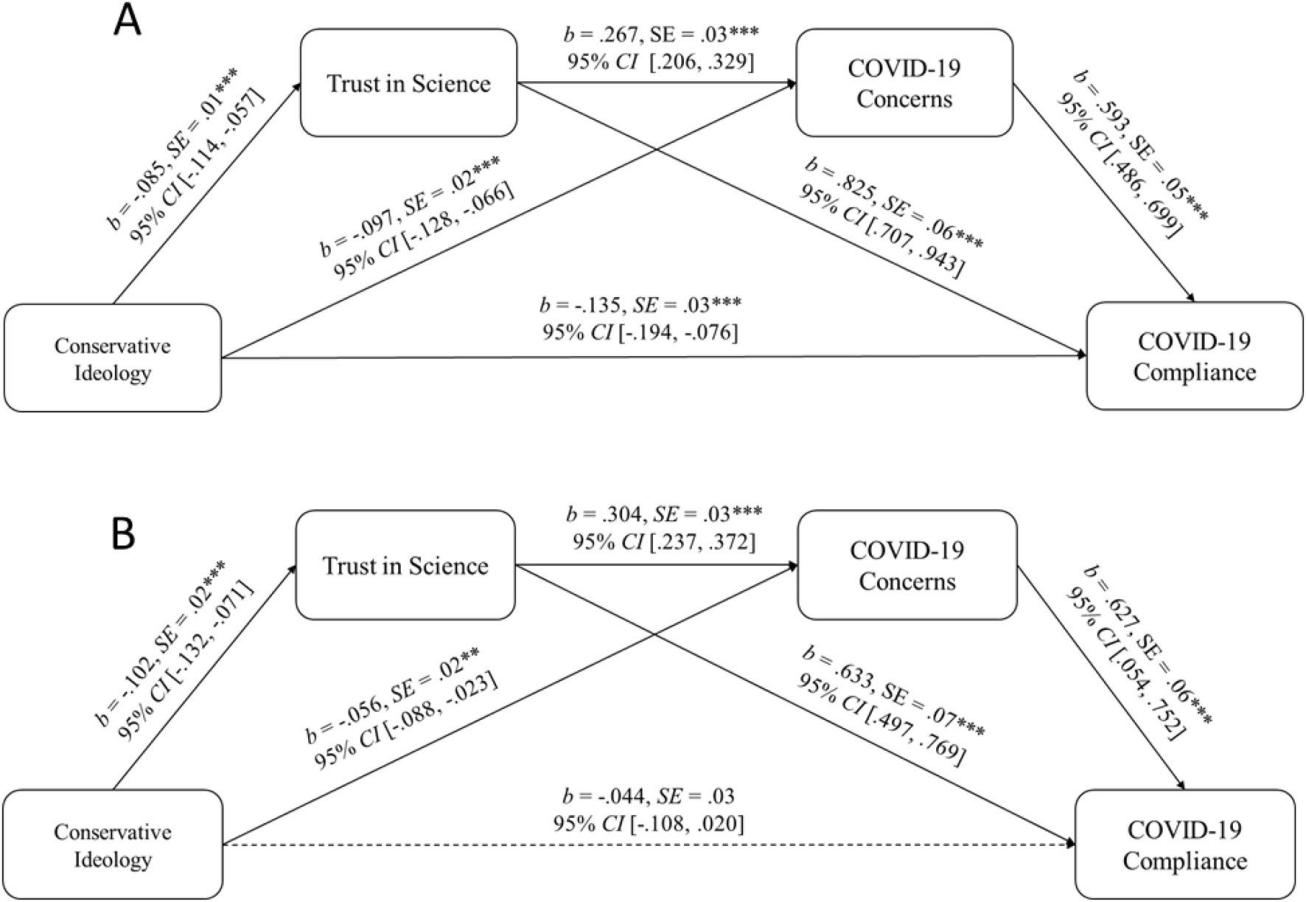
(**A**, **B**). Indirect effect tests (Process Macro, Model 6^55^, 10,000 bootstrapped samples) of conservative ideology on compliance to COVID-19 recommendations, via the indirect sequential pathways of trust in science and concerns about COVID-19, controlling for maleness (male = 1, not male = − 1), income, education level, and age, in both Study 2a (**A**, top) and 2b (**B**, bottom). Figure was constructed in Microsoft PowerPoint with manually input data copied from SAS 9.4 output. *Note:* **p < .01; ***p < .001.

To test our full hypothesis in each study, we computed an indirect effect test. We controlled for age, being male (compared to being either female or non-binary, male = 1; not male = − 1; analyzed thusly as there is evidence that men are less likely to comply with recommended behaviors than other genders^56,57^ income and education level. Results across both studies suggested that conservative ideology was associated with both less trust in science and less concern about the pandemic. Both trust in science and concerns about COVID-19, in turn, were associated with greater compliance. All indirect effects were significant across both studies (Table 2). While the direct effect of conservatism upon compliance remained significant before the 2020 Presidential election (Study 2a), the direct effect was non-significant after the election (Study 2b; Fig. 2A–B).

**Table 2:** Indirect effects depicted in Fig. 2a–b.

	Study 2a					Study 2b				
	Effect	SE	Lower 95% CI	Upper 95% CI	% of Total Effect	Effect	SE	Lower 95% CI	Upper 95% CI	% of Total Effect
Total Indirect effect	− 0.14	0.02	− 0.18	− 0.10	50.65	− 0.12	0.02	− 0.16	− 0.08	77.92
Ideology→ Trust in Science→ Compliance	− 0.07	0.01	− 0.1	− 0.04	25.33	− 0.06	0.01	− 0.09	− 0.04	38.96
Ideology→Concerns→Compliance	− 0.06	0.01	− 0.08	− 0.04	21.71	− 0.03	0.01	− 0.06	− 0.01	19.48
Ideology→ Trust in science→Concerns→Compliance	− 0.01	0.004	− 0.02	− 0.008	3.62	− 0.02	0.01	− 0.03	− 0.01	12.99

## Study 3

In Study 3, we sought to re-examine our hypothesis across different nations, once again employing representative samples. Our aim was to determine whether the negative link between a conservative political ideology with trust in science and beliefs towards the Coronavirus pandemic (i.e. concern about the pandemic, compliance with COVID-19 recommendations, and support for stricter measures to prevent the spread of the pandemic), is found across nations, or is localized to particular countries, such as the United States, given the relationships between conservative U.S. media ecosystems and coronavirus responses (see^7^).

## Methods

### Participants

We recruited data across 21 countries and special administrative regions, recruiting a total of 25,159 participants. These included: Australia, Canada, China, Spain, France, Germany, Hong Kong, Hungary, Indonesia, the Republic of Ireland, Israel, Italy, Korea, Malaysia, the Netherlands, the Philippines, Poland, Serbia, Turkey, the United Kingdom, and the United States. In China and Hong Kong no measure of political ideology was included in the survey because of risks to participants, and thus participants from these countries were excluded from the current investigation, leaving us with a total of 19 countries (*N* = 20,580; Table S2 for country specific *N*s and descriptive statistics). Participants were recruited across three cross-sectional waves: Wave 1: May 4th, 2020–May 21st, 2020; Wave 2: June 15th, 2020–June 23rd, 2020; Wave 3: July 20th, 2020–July 28th, 2020, via CloudResearch^58,59^. Samples sizes were determined based on an a priori power analysis detect interactions between time-point comparisons and cross-country comparisons for a small-to-medium effect size (Cohen’s *f* = 0.160; see^60^). Thus, we aimed to recruit at least 300 participants per wave in each country. To approach a more representative sample from each country, data was collected to fill known representative percentages for a variety of demographic characteristics, such as level of education, race/ethnicity, urbanization, religion, age, gender, income. These levels were established through census-level data of each country population. In the U.S we aimed to recruit truly representative samples, via the same demographic characteristics as above but with a larger number of participants (*N* = 1200) per wave. While these analyses were not pre-registered, the sampling method was pre-determined for all samples (see https://osf.io/g29z4/). All research conducted in this study received approval from the University of Massachusetts Amherst IRB (IRB Protocol #2063) and complied with all APA guidelines and policies for human subjects research. Informed consent was obtained by the researchers for all participant data used in Study 3.

### Materials and procedure

Participants first provided consent, and then completed a questionnaire with various measures. After completing all the measures and providing demographic information, participants were asked questions pertaining to any upcoming or recently concluded elections in their country, and were then subsequently debriefed and remunerated. All measures were first generated in English. They were then translated/back-translated into applicable languages for each country. In the sections that follow, descriptive statistics and reliabilities capture values across all waves and countries (Table S2 for country-specific information). Unless otherwise noted, all measures were captured on a 1–9 slider scale.

#### Political ideology

An item identical to that utilized in Studies 2a and 2b, measured on a 1–7 Likert scale was used to capture left/right wing ideology (*M* = 3.80, *SD* = 1.57).

#### Trust in science

We generated two items to measure trust in science, which preceded by the sentence: “To what extent do you trust information about coronavirus if it comes from each of the following information sources?” (“Scientific authorities and professionals (e.g., epidemiologists, virologists)” and “Medical professionals (e.g., doctors, nurses, surgeons, EMTs)”). Trust in science was measured with the average of these two items (*a* = 0.77, *α*_*range*_ = 0.68–0.82, *M* = 7.11, *SD* = 1.64).

#### Concerns about contracting COVID-19

A single-item measure (“Which, if any, of the following statements describes your feelings toward getting the coronavirus? I am not at all/not very/ somewhat/ very scared I will contract the coronavirus (COVID-19)”); this measure was identical to one of three items used to capture COVID-19 concerns in Studies 2a and 2b). This measure was captured on a 1–4 Likert scale (*M* = 2.49, *SD* = 1.10).

#### Compliance with coronavirus guidelines

A four-item measure was developed to capture the degree to which participants complied with scientifically-recommended coronavirus (COVID-19) guidelines to reduce the infection of the virus (“How often do you wash your hands with soap and water for at least 20 s when you enter or exit your home?”; “How often do you stay at least 6 feet (or 2 m) away from anyone who is not a member of your household when you are outside your home (e.g., social distancing?)”; “Do you avoid social gatherings due to the coronavirus?”; “Have you been cancelling, and are you avoiding, any non-essential travel”). The measure was overall reliable (*a* = 0.77, *α*_*range*_ = 0.61–0.83, *M* = 7.49, *SD* = 1.51).

#### Support for lockdown restrictions

Seven-items were generated to measure how much participants supported preventative restrictions upon civil liberties during lockdowns (e.g., “National intelligence services should track and collect data from people suspected to be infected with coronavirus;” “The military should be used domestically in order to assist with responses to the coronavirus”). This measure was reliable as well (*a* = 0.84, *α*_*range*_ = 0.76–0.89, *M* = 6.17, *SD* = 1.80).

### Data analysis

For preliminary analyses, we estimated correlations within each country for each wave between political ideology and: (1) trust in science, (2) concerns about contracting COVID-19, (3) compliance with COVID-19 regulations, (4) support for lockdown restrictions to prevent the spread of the virus using the *proc corr* command in SAS 9.4^53^. These correlations were used in order to estimate mean correlation effect sizes (“meta-correlations”) across all three cross-sectional waves in each country using Goh and colleagues’ (2016) methodology and publicly available calculation spreadsheets (see https://osf.io/8yubf/). These publicly available materials detail the exact mathematical procedures used to compute and test the significance of meta-correlations. Thus, our meta-correlations were computed by inputting our data into these Microsoft Excel spreadsheets. All of the above procedures were repeated in order to evaluate the correlations and meta-correlations between trust in science and (1) concerns about contracting COVID-19, (2) compliance with COVID-19 regulations, and (3) support for lockdown restrictions.

To replicate the indirect effects observed in the previous studies, while adding support for lockdown restrictions as an additional outcome, we constructed a series of path models in SAS 9.4^53^ using the *proc calis* command. Using the *proc calis* syntax, we allowed all exogenous variables to predict both sequential mediators (i.e., trust in science, concerns about contracting COVID-19), and both sequential mediators to predict both outcomes (compliance and support for lockdown restrictions), yielding a fully saturated model (see Fig. 4 for diagram). We constructed one path model for each country in our sample. We then compared the path from political ideology to trust in science in the U.S. and Canada (separately) to each other and to each of the 17 other countries in our sample.

### Ethical statement

All data collection procedures in which the authors collected data from human subjects were approved by the University of Massachusetts Amherst Institutional Review Board (IRB). Separate protocols were approved for Studies 2a–2b (IRB Protocol #2317) and Study 3 (IRB Protocol #2063). All procedures were performed in accordance with the guidelines and regulations for human subjects research set by the University of Massachusetts Amherst IRB. Informed consent was obtained from all participants.

## Results

### Meta-Correlations

#### Political ideology

**Figure 3:**
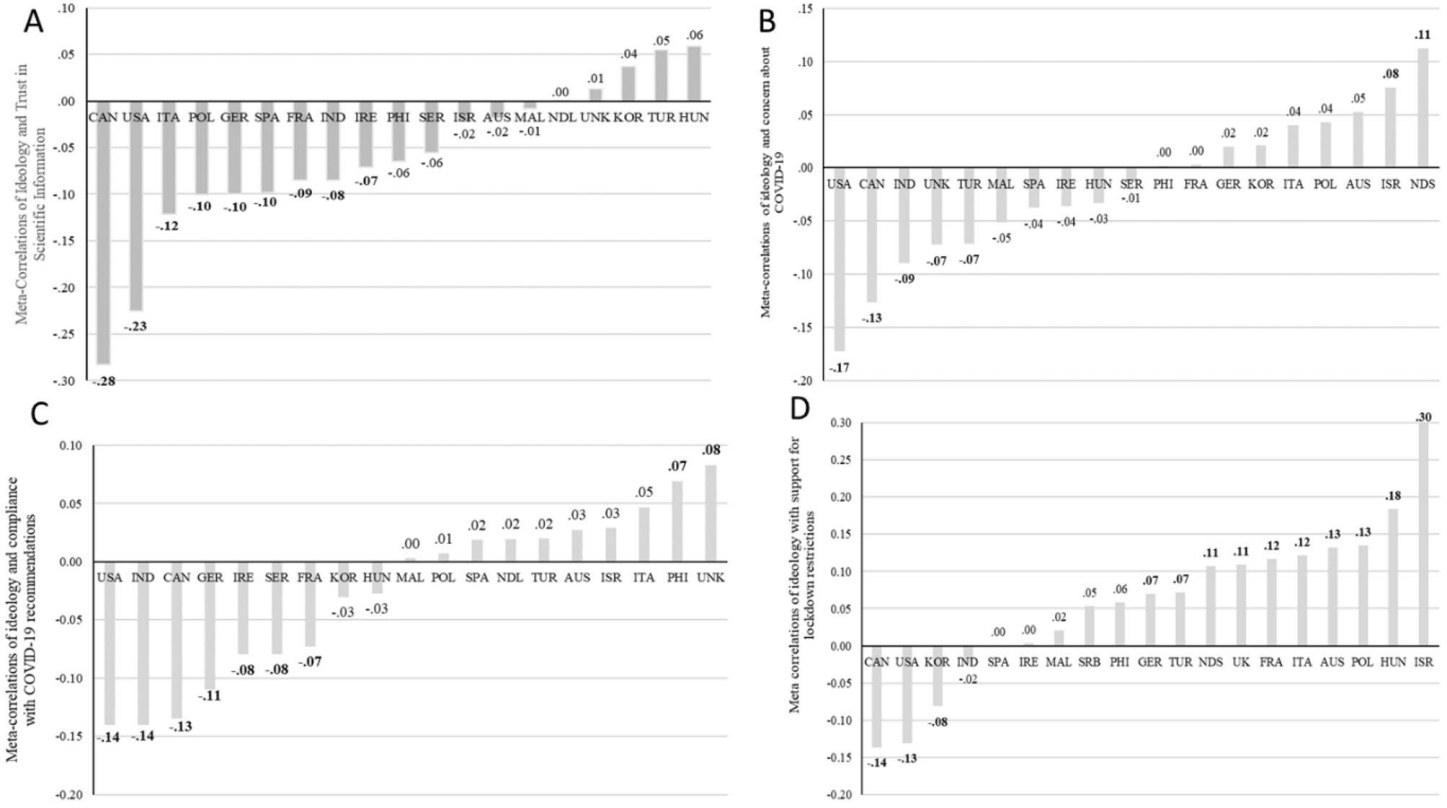
Visual depiction of meta-correlations between conservative/right wing political ideology and (**A**) trust in scientific information about coronavirus across the three cross-sectional waves for each country; (**B**) concern about contracting the coronavirus across the three cross-sectional waves for each country; (**C**) compliance with recommended preventative behaviors to avoid contracting or spreading the coronavirus across the three cross-sectional waves for each country; (**D**) support for lockdown restrictions across the three cross-sectional waves for each country. Figures were constructed in Microsoft Excel using the same spreadsheet in which the meta-correlations were generated. *Note:* Bolded values depict significant meta-correlations for all sub-figures. Values > 0.08 are significant at *p* < 0.05, values > 0.09 are significant at *p* < 0.01, and values > 0.10 are significant at *p* < 0.001.

Correlations between political ideology and other variables in the model in each wave and in each country and their corresponding meta-correlations are summarized in Supplementary Materials (raw correlations: Tables S3–S6; meta-correlations: Tables S7–S10). The meta-correlations are visualized in Fig. 3A–D. The United States and Canada alone exhibited significant negative meta-correlations between conservative ideology and all outcome variables. For trust in science and concern about contracting coronavirus, their meta-correlations were also larger than those of the other 17 countries (Fig. 3A–B). Also, while there was no significant relationship between conservatism and trust in science in 10 of 19 countries, a positive meta-correlation was never observed (Fig. 3A). However, for compliance, Indonesia and Germany exhibited meta-correlations of a similar strength to those of the U.S. and Canada, although the U.S. and Canada were still among the largest across countries (Fig. 3C). With respect to lockdown restrictions, only three countries demonstrated a negative meta-correlation with conservatism: The United States, Canada, and South Korea, whereas the meta-correlations were positive for 10 of 19 countries, with Israel’s being the strongest (Fig. 3D).

#### Trust in science

Raw correlations between trust in science and other variables in the model are presented in Supplementary Materials in Table S11. The meta-correlations we found suggest that while direct associations between conservatism and coronavirus responses might be localized to particular countries, links between trust in scientific information and outcomes are more consistent. Meta-correlations between trust in scientific information about coronavirus and concern about contracting the coronavirus were significant and positive in 16 of 19 countries (*r*_median_ = 0.12, *r*_min_ = 0.07, *r*_max_ = 0.20, all *p*s < 0.05), and were never significant and negative (Figure S1). Further, both the meta-correlation between trust in scientific information about coronavirus and compliance with preventative behaviors (*r*_median_ = 0.35, *r*_min_ = 0.22, *r*_max_ = 0.47, all *p*s < 0.001; Figure S2) and the meta-correlation between trust in scientific information about coronavirus and support for lockdown restrictions (*r*_median_ = 0.29, *r*_min_ = 0.15, *r*_max_ = 0.44, all *p*s < 0.001, Figure S3) were significant and positive in all countries. These meta-correlations suggest that while the role of conservatism and trust in scientific information may vary across countries, the subsequent relationships between that trust and outcome variables are consistent across countries. Therefore, the association between conservatism and these outcomes could be mediated by trust in scientific information, so long as an association between conservatism and trust in scientific information about the coronavirus is observed.

### Multigroup path analysis

The results of our path analyses suggested that the association between political ideology and trust in scientific information about the coronavirus did not differ between the U.S. and Canada but was significantly stronger in the United States for 15 out of the 17 countries and in Canada for 13 out of the 17 countries (Table 3). Further evidence in these models suggest that this association is strongest in North America, with one exception (Indonesia). The indirect effect of political ideology on concerns about contracting COVID-19 (i.e., ideology→trust in science→COVID-19 concern) was only significant in one other country (Italy); as was the indirect effect for compliance (i.e., ideology→trust in science→COVID-19 concern→COVID-19 compliance; in Spain), and the indirect effect for support for lockdown restrictions (i.e., ideology→trust in science→COVID-19 concern→lockdown restrictions) was only significant in two other countries (Spain, the Netherlands), one of which exhibited an effect in the opposite direction (the Netherlands).

**Table 3:** Indirect effects of political ideology on each variable, and multigroup comparisons for the association between ideology and trust in science, from Fig. 4.

Country	Path a comparisons: United States	Path a comparisons: Canada	Ideology → Trust Science	Indirect effect on Concern	% of total effect	Indirect effect on Compliance	% of total effect	Indirect effect on Curtailments	% of total effect
United States	–	*b* = − 0.01, SE = 0.03, *t* = − 0.40, *p* = 0.692	− 0.19 (0.01)***	− 0.02 (.01)***	22%	− 0.09 (.01)***	66%	− 0.09 (.01)***	61%
Canada	*b* = 0.01, SE = 0.03, *t* = 0.40, *p* = 0.692	–	− 0.20 (0.03)***	− 0.01 (.01)*	19%	− 0.10 (.02)***	NA	− 0.08 (.01)***	NA
Australia	***b* = − 0.15, *SE* = 0.03, *t* = − 4.21, *p* < 0.001**	***b* = − 0.17, *SE* = 0.05, *t* = − 3.27, *p* = 0.001**	n.s	n.s		n.s		n.s	
Spain	***b* = − 0.07, *SE* = 0.03, *t* = − 2.17, *p* = 0.030**	*b* = − 0.08, *SE* = 0.04, *t* = − 1.93, *p* = 0.053	− 0.11 (0.03)***	n.s		− 0.04 (0.01)**	NA	− 0.04 (0.01)***	NA
France	***b* = − 0.13, *SE* = 0.04, *t* = − 3.29, *p* = 0.001**	***b* = − 0.14, *SE* = 0.05, *t* = − 3.00, *p* = 0.002**	n.s	n.s		n.s		n.s	
Germany	***b* = − 0.09, *SE* = 0.04, *t* = − 2.38, *p* = 0.017**	*b* = − 0.11, *SE* = 0.05, *t* = − 1.92, *p* = 0.054	n.s	n.s		n.s		n.s	
Hungary	***b* = − 0.26, *SE* = 0.04, *t* = − 6.41, *p* < 0.001**	***b* = − 0.27, *SE* = 0.05, *t* = − 5.28, *p* < 0.001**	n.s	n.s		n.s		n.s	
Indonesia	*b* = − 0.05, *SE* = 0.03, *t* = − 1.77, *p* = 0.076	*b* = − 0.07, *SE* = 0.04, *t* = − 1.51, *p* = 0.131	− 0.12 (0.03)***	− 0.01 (.01)*	14%	− 0.05 (.01)***	26%	− 0.05 (0.01)***	NA
Ireland	***b* = − 0.11, *SE* = 0.03, *t* = − 3.24, *p* = 0.001**	***b* = − 0.13, *SE* = 0.05, *t* = − 2.56, *p* = 0.010**	n.s	n.s		n.s		n.s	
Israel	***b* = − 0.12, *SE* = 0.03, *t* = − 3.36, *p* < 0.001**	***b* = − 0.13, *SE* = 0.05, *t* = − 2.78, *p* = 0.005**	n.s	n.s		n.s		n.s	
Italy	***b* = − 0.09, *SE* = 0.04, *t* = − 2.35, *p* = 0.018**	***b* = − 0.10, *SE* = 0.05, *t* = − 2.14, *p* = 0.032**	− 0.10 (0.04)**	− 0.01 (0.01)*	NA	n.s		n.s	
South Korea	***b* = − 0.23, *SE* = 0.03, *t* = − 7.57, *p* < 0.001**	***b* = − 0.24, *SE* = 0.04, *t* = − 5.35, *p* < 0.001**	n.s	n.s		n.s		n.s	
Malaysia	***b* = − 0.14, *SE* = 0.03, *t* = − 4.36, *p* < 0.001**	***b* = − 0.16, *SE* = .05, *t* = − 3.36, *p* < .001**	n.s	n.s		n.s		n.s	
Netherlands	***b* = − 0.18, *SE* = 0.03, *t* = − 5.18, *p* < 0.001**	***b* = − 0.20, *SE* = 0.05, *t* = − 3.95, *p* < 0.001**	n.s	n.s		n.s		0.03 (0.02)*	NA
Philippines	***b* = − 0.13, *SE* = 0.03, *t* = − 4.09, *p* < 0.001**	***b* = − 0.14, *SE* = 0.04, *t* = − 3.15, *p* = 0.002**	n.s	n.s		n.s		n.s	
Poland	***b* = − 0.11, *SE* = 0.04, *t* = − 3.06, *p* = 0.002**	***b* = − 0.12, *SE* = 0.05, *t* = − 2.53, *p* = 0.011**	n.s	n.s		n.s		n.s	
Serbia	*b* = − 0.09, *SE* = 0.04, *t* = − 1.94, *p* = 0.052	*b* = − 0.10, *SE* = 0.06, *t* = − 1.70, *p* = 0.088	n.s	n.s		n.s		n.s	
Turkey	***b* = − 0.24, *SE* = 0.04, *t* = − 6.47, *p* < 0.001**	***b* = − 0.26, *SE* = 0.05, *t* = − 5.29, *p* < 0.001**	n.s	n.s		n.s		n.s	
United Kingdom	***b* = − 0.20, *SE* = 0.03, *t* = − 5.81, *p* < 0.001**	***b* = − 0.21, *SE* = 0.04, *t* = − 4.68, *p* < 0.001**	n.s	n.s		n.s		n.s	

*p < 0.05, **p < 0.01, ***p < 0.001, n.s. = not significant. The USA and Canada were the reference group in each comparison. Bold values depict significant results. Calculations of the proportion of the total effect calculated by the indirect effect were made by dividing the indirect effect of trust in science by the total effect. If the total effect was not significant or in the opposite direction, the calculation was not feasible, and thus NA (Not Available) is indicated).

**Figure 4:**
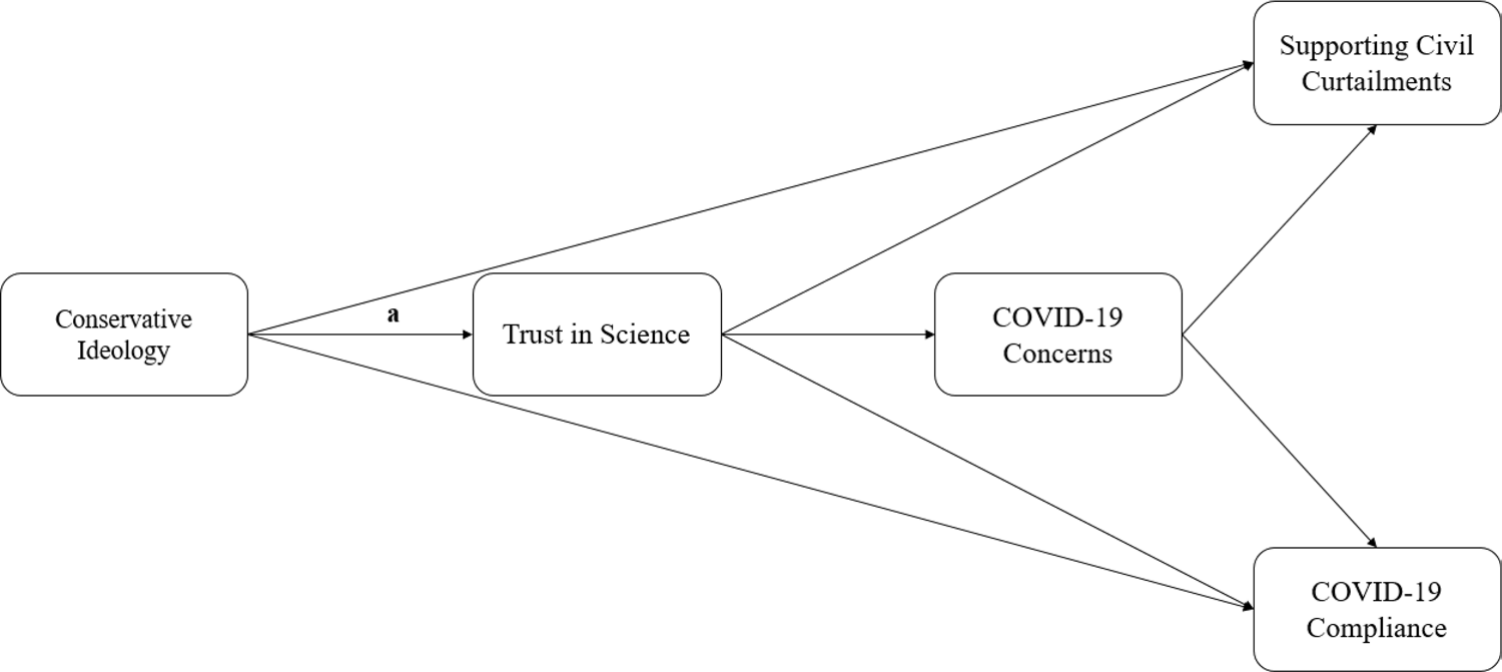
Path Model tested in each of the 19 countries. Path a is the path for which multigroup comparisons were estimated. Figure was constructed in Microsoft PowerPoint.

## Discussion

Across three studies, we found evidence that among Americans, conservatism is associated with less compliance with recommended preventative behaviors to avoid coronavirus infection, sequentially mediated through trust in scientific information sources and concern about the threat of personal infection by coronavirus. Studies 1a–1b found and replicated evidence for indirect effects of conservatism on concern about contracting coronavirus through trust in science in two representative samples of the U.S. population before and after the 2020 U.S. presidential election. Studies 2a–2b replicated these findings with independent representative samples of the U.S. population, and found evidence that this indirect effect sequentially extends to compliance with preventative behaviors. Study 3 further replicates these patterns among representative samples of Americans, and expands the model by adding support for lockdown restrictions as another outcome. Such findings are consistent with other studies of American populations see^4–7^.

Evidence was more mixed, however, for the hypothesis that the indirect effects we observed would be strongest in, or unique to, the United States. The sequential indirect effect model in the U.S. was found in only two other countries in our sample—Canada and Indonesia. Divergent meta-correlations between conservatism and other variables in the model, particularly trust in scientific information about the coronavirus, appear to be driving the non-replication of the model outside of these three countries. Across the countries sampled, trust in scientific information about the coronavirus was consistently correlated with concern about contracting the virus, compliance, and support for lockdown restrictions (Figures S1–S3), suggesting that its importance is generalizable^16^. Yet, meta-correlations between conservatism and rust in scientific information about coronavirus, concern about contracting coronavirus, self-reported behavior compliance, and support for lockdown restrictions were consistently stronger in the U.S. and Canada compared to other countries. Further, meta-correlations of similar magnitude consistently emerged for Indonesia. Thus, while the U.S. did indeed demonstrate stronger patterns than most other nations, it did not do so uniquely.

That Canadians exhibited similar responses to Americans is partly explainable through its proximity to, and shared media ecosystem with, the United States see^61^ and indeed, misinformation about the coronavirus in Canadian social media appears to originate from U.S. media^62^. The same pattern emerging in Indonesia, however, cannot logically be explained by media overlap with the United States. Rather, the emergence of similar (but weaker) indirect effects in Indonesia may be explainable by a similarly extreme polarization around trust in the scientific consensus, within the context of the coronavirus pandemic^63^.

Regardless, one broader implication of these findings is that across the countries we sampled from, conservatism neither universally nor necessarily predicts non-compliant coronavirus behaviors or even lack of concern in and of itself. While care should be used comparing meta-correlations for conservatism across countries, as its precise meaning can vary cross-nationally^64^ and across time and cultural groups^65,66^, evidence of political groups clustering along a left–right axis can be used to make cautious statements of patterns see^66–68^. Although there are psychological factors associated with conservatism that may predispose conservatives to generally distrust science more [see^28,30^], our results suggest that this association is not an inevitability across the globe, but rather, emergent from specific national contexts. That we never found conservatism to positively predict trust in scientific information about coronavirus, even in Israel, where conservative leadership took a hard line during the pandemic, suggests a tendency may still be present. It is also worth noting that conservatism was, in many countries, positively associated with lockdown restrictions, perhaps resonating with authoritarian tendencies [see^30^]. That said, the results from the U.S., Canada, and Indonesia also clearly suggest that, under the right circumstances, liberals can be more sensitive to certain threats than conservatives, in line with multidimensional approaches to understanding ideology and threat^13,38,39^.

The results we observe suggest that part of how political ideology relates to threat perceptions, however multidimensional, operates through trust in information about the relevant threat—in this case, the threat of personal infection with coronavirus. While conservatives and liberals both *can* be more or less sensitive to threats depending on the type of threat (commission vs omission, see^14^), what our results suggest is that distrust in warnings about the threat may lessen the degree to which the threat is perceived at all. However, it should be noted that such associations need not inevitably lead to disaster. That Canada and Indonesia, despite sharing the patterns found in the U.S., did not experience the same degree of catastrophic losses from the coronavirus pandemic as the United States hints that, individual differences aside, early, unified action by political elites and systemic preparation may help protect against the negative effects instantiated by low trust in science [see^69^], a possibility supported by findings that countries with earlier, more restrictive pandemic responses have fared better thus far [see^70–72^]. Further, the positive meta-correlations we observe between conservatism and support for lockdown restrictions in many of the countries sampled implies that, perhaps owing to overlap between conservatism and authoritarianism, conservatives will support such measures if they are not positioned as contradictory to their ingroup by partisan media (as in the U.S. and Canada, where the meta-correlations were negative).

The most important limitation of these data and analyses is that they are observational (i.e., correlational), and thus, causality cannot be inferred, despite arguments across recent literature for the directionality used in our models (see^16–18^). Further, it has been argued, and demonstrated, that regression-based mediation models based on correlational data can be prone to biased effect sizes when variable error terms are correlated (see^73^). While we have taken steps to avoid this possibility, including using bootstrapping and path modelling rather than traditional Baron-Kenney mediation, Bullock and colleagues (2010) note that even with best practices, this possibility cannot be completely disregarded. Nevertheless, Hayes (2017) notes that the bootstrapping procedures are typically acceptable with correlational data so long as the links tested are theoretically sound, and the models we tested were grounded in emergent literature (see^16–18^). Further, at least within the United States, links between conservatism and trust in science more generally are reflecting in existing evidence^15,40–44^.

A second limitation of these studies is that while they examine *conservatism*, as understood by participants, they conceptualize conservatism as a unitary construct^60^ and they do not examine ideological factors among conservatives that might have divergent effects upon attitudes and behaviors within the coronavirus pandemic, such as right-wing authoritarianism (RWA; see^35–37^) or social dominance orientation (SDO; see^74,75^). While we did not find, for example, a relationship between conservatisvm and concern about coronavirus infection in our Australian samples in Study 3, Clarke et al. (2021) found relationships between some specific dimensions of SDO and RWA^76^, such that they predicted less concern about contracting the coronavirus, similar to the associations with RWA in American samples^77^. Our single-item measure of unitary conservatism cannot distinguish between laissez-faire conservatism (sometimes called *economic conservatism*) and authoritarian conservatism (sometimes called *social conservatism*) [see^78^]. Because we measured a unitary construct of conservatism, participants could have different understandings of “conservatism,” both within and between countries. These limitations are particularly important for understanding the positive meta-correlations between conservatism and support for lockdown restrictions that we observe in the majority of countries that we sampled. Thus, further research should examine more specific elements and dimensions of ideology as well as high-level endorsement of “conservatism” or “liberalism.”

In a similar vein, our findings are limited in that we cannot directly test *why* conservatism is negatively associated with trust in scientific information about the coronavirus pandemic in some countries, most notably the United States and Canada. While existing literature on general scientific polarization (for a review, see^5^) would suggest that conservatism may be linked to negative views of scientists and scientists as politicized, antagonistic outgroups (e.g., liberals, elites), our studies do not measure perceptions of scientists as a group. Another possibility, suggested by Gollwitzer and colleagues’ findings (2020) is that politicized media ecosystems play a role in shaping such relationships. Future research should investigate the role such factors play in the associations that we have observed here. Lastly, future research should also endeavor to gather information on these associations in South America, Africa, India, and other parts of the globe we were unable to reach.

## Conclusion

Here, three studies reinforce links between conservatism and attitudes and behaviors during the coronavirus among Americans, while also explicitly highlighting the role that trust in science and scientific information sources about the pandemic plays in these processes. Apart from conceptually replicating and extending existing research on Americans, Study 3 suggests that Canadians and Indonesians may also exhibit the same polarization of attitudes through trust in scientific information about the coronavirus. These results suggest that, rather than conservatism per se inevitably leading to skepticism about pandemics, the emergence of such a link is contextual, similar to prior findings regarding polarizing issues such as climate change skepticism^42^. Therefore, management of future pandemics may hinge upon how well scientific communicators can manage the contextual framing of the pandemic as it arises, lest the particularly disastrous patterns observed in places like the United States be repeated.

## Supplementary Information

Supplementary Information.

## Data Availability

For all studies, materials, data, and analysis code are available here: [https://osf.io/ugde5/?view_only=dc4c3b9d8a79433cab5a2fbc9e663a1d].

## References

[1] Johns Hopkins University (2021). Coronavirus Resource Center. Accessed from: https://coronavirus.jhu.edu/map.html.

[2] Nuzzo JB, Bell JA, Cameron EE (2020). Suboptimal US response to COVID-19 despite robust capabilities and resources. JAMA.

[3] Van Bavel J, Baicker K, Boggio PS, Capraro V, Cichocka A, Cikara M, Crockett MJ, Crum AJ, Douglas KM, Druckman JN, Drury J, Dube O, Ellemers N, Finkel EJ, Fowler JH, Gelfand M, Han S, Haslam SA, Jetten J, Kitayama S (2020). Using social and behavioural science to support COVID-19 pandemic response. Nat. Hum. Behav..

[4] Calvillo DP, Ross BJ, Garcia RJB, Smelter TJ, Rutchick AM (2020). Political ideology predicts perceptions of the threat of COVID-19 (and susceptibility to fake news about it). Soc. Psychol. Personal. Sci..

[5] Christensen SR, Pilling EB, Eyring JB, Dickerson G, Sloan CD, Magnusson BM (2020). Political and personal reactions to COVID-19 during initial weeks of social distancing in the United States. PLoS ONE.

[6] Latkin CA, Dayton L, Moran M, Strickland JC, Collins K (2021). Behavioral and psychosocial factors associated with COVID-19 skepticism in the United States. Curr. Psychol..

[7] Gollwitzer A, Martel C, Brady WJ (2020). Partisan differences in physical distancing are linked to health outcomes during the COVID-19 pandemic. Nat. Human Behav..

[8] Jost JT, Stern C, Rule NO, Sterling J (2017). The politics of fear: Is there an ideological asymmetry in existential motivation?. Soc. Cogn..

[9] Thornhill R, Fincher CL, Aran D (2009). Parasites, democratization, and the liberalization of values across contemporary countries. Biol. Rev. Camb. Philos. Soc..

[10] Matthews M, Levin S, Sidanius J (2009). A longitudinal test of the model of political conservatism as motivated social cognition. Polit. Psychol..

[11] Crawford JT (2017). Are conservatives more sensitive to threat than liberals? It depends on how we define threat and conservatism. Soc. Cogn..

[12] Bakker BN, Schumacher G, Gothreau C, Arceneaux K (2020). Conservatives and liberals have similar physiological responses to threats. Nat. Hum. Behav..

[13] Brandt MJ, Turner-Zwinkels FM, Karapirinler B, Van Leeuwen F, Bender M, van Osch Y, Adams B (2021). The association between threat and politics depends on the type of threat, the political domain, and the country. Pers. Soc. Psychol. Bull..

[14] Kahn, D. T., Björklund, F., & Hirschberger, G. (2021). The intent and nature of collective threats: A data-driven conceptualization of collective threats and their relation to political preferences. *J. Exp. Psychol. General*. Accepted Manuscript. 10.1037/xge0000868 34843368

[15] Rekker R. The nature and origins of political polarization over science. Public Understanding of Science, Advance Online Publication. (2021) 10.1177/0963662521989193 10.1177/0963662521989193 PMC8114456 33594929

[16] Plohl, N., & Musil, B. Modeling compliance with covid-19 prevention guidelines: The critical role of trust in science. *Psychol. Health Med.* (2020) Advance online publication. 10.1080/13548506.2020.1772988 10.1080/13548506.2020.1772988 32479113

[17] Sulik, J., Deroy, O., Dezecache, G., Newson, M., Zhao, Y., El Zein, M., & Tuncgenc, B. (2021, March 4). Facing the pandemic with trust in science. 10.31234/osf.io/edw47

[18] Pagliaro S, Sacchi S, Pacilli MG, Brambilla M, Lionetti F (2021). Trust predicts COVID-19 prescribed and discretionary behavioral intentions in 23 countries. PLoS ONE.

[19] Pennycook, G., McPhetres, J., Bago, B., & Rand, D. G. (2020, April 14). Beliefs about COVID-19 in Canada, the U.K., and the U.S.A.: A novel test of political polarization and motivated reasoning. 10.31234/osf.io/zhjkp 10.1177/01461672211023652 PMC9066691 34180276

[20] McCright AM, Dunlap RE (2010). Anti-reflexivity. Theory Cult. Soc..

[21] Azevedo F, Jost JT (2021). The ideological basis of antiscientific attitudes: Effects of authoritarianism, conservatism, religiosity, social dominance, and system justification. Group Process. Intergroup Relat..

[22] McCright AM, Dentzman K, Charters M, Dietz T (2013). The influence of political ideology on trust in science. Environ. Res. Lett..

[23] Rutjens BT, Sutton RM, van der Lee R (2018). Not all skepticism is equal: exploring the ideological antecedents of science acceptance and rejection. Pers. Soc. Psychol. Bull..

[24] Nisbet EC, Cooper KE, Garrett RK (2015). The partisan brain: how dissonant science messages lead conservatives and liberals to (dis)trust science. Ann. Am. Acad. Pol. Soc. Sci..

[25] Proulx T, Brandt MJ (2017). Beyond threat and uncertainty: The underpinnings of conservatism. Soc. Cogn..

[26] Washburn AN, Skitka LJ (2018). Science denial across the political divide: Liberals and conservatives are similarly motivated to deny attitude-inconsistent science. Soc. Psychol. Person. Sci..

[27] Gauchat G (2012). Politicization of science in the public sphere: a study of public trust in the United States 1974 to 2010. Am. Sociol. Rev..

[28] Mooney C (2012). The Republican brain: the science of why they deny science–and reality.

[29] Nash GH (2014). The conservative intellectual movement in America since 1945.

[30] Dunlap RE, McCright AM, Yarosh JH (2016). The political divide on climate change: Partisan polarization widens in the U.S. Environment. Sci. Policy Sustain. Dev..

[31] Mildenberger M, Marlon JR, Howe PD, Leiserowitz A (2017). The spatial distribution of Republican and Democratic climate opinions at state and local scales. Clim. Change.

[32] Oreskes N, Conway EM (2011). Merchants of doubt: how a handful of scientists obscured the truth on issues from Tobacco Smoke to global warming.

[33] Carmichael JT, Brulle RJ, Huxster JK (2017). The great divide: understanding the role of media and other drivers of the partisan divide in public concern over climate change in the USA, 2001–2014. Clim. Change.

[34] Hornsey MJ, Harris EA, Fielding KS (2018). Relationships among conspiratorial beliefs, conservatism, and climate skepticism across nations. Nat. Clim. Change.

[35] Adorno TW, Frenkel-Brunswik E, Levinson DJ, Sanford RN (1950). The Authoritarian Personality.

[36] Altemeyer B (1988). Enemies of freedom: understanding right-wing authoritarianism.

[37] Duckitt J, Sibley CG (2010). Personality, ideology, prejudice, and politics: a dual-process motivational model. J. Personal..

[38] Hirschberger G, Ein-Dor T, Leidner B, Saguy T (2016). How is existential threat related to intergroup conflict? introducing the multidimensional existential threat (MET) model. Front. Psychol..

[39] Eadeh FR, Chang KK (2020). Can threat increase support for liberalism? New insights into the relationship between threat and political attitudes. Soc. Psychol. Person. Sci..

[40] McCright A (2010). The effects of gender on climate change knowledge and concern in the American public. Popul. Environ..

[41] Feldman L, Sol Hart P, Milosevic T (2017). Polarizing news? representations of threat and efficacy in leading US newspapers’ coverage of climate change. Public Underst. Sci..

[42] Dunlap RE, McCright AM, Dryzek J, Norgaard R, Schlosberg D (2011). Organized climate change denial. Oxford Handbook of Climate Change and Society.

[43] Douglas H (2015). Politics and science: untangling values, ideologies, and reasons. Ann. Am. Acad. Pol. Soc. Sci..

[44] Ruisch BC, Moore C, Granados Samayoa J, Boggs S, Ladanyi J, Fazio R (2021). Examining the left-right divide through the lens of a global crisis: ideological differences and their implications for responses to the COVID-19 pandemic. Polit. Psychol..

[45] Summers, J. Timeline: How Trump Has Downplayed The Coronavirus Pandemic. *NPR*. https://www.npr.org/sections/latest-updates-trump-covid-19-results/2020/10/02/919432383/how-trump-has-downplayed-the-coronavirus-pandemic (2020, Oct 2)

[46] Keith, T. Trump Says He Downplayed Coronavirus Threat In U.S. To Avert Panic. *NPR.* https://www.npr.org/2020/09/11/911828384/trump-says-he-downplayed-coronavirus-threat-in-u-s-to-avert-panic (2020, Sept 11)

[47] Peters, J. W. Alarm, Denial, Blame: The Pro-Trump Media’s Coronavirus Distortion. *The New York Times.* https://www.nytimes.com/2020/04/01/us/politics/hannity-limbaugh-trump-coronavirus.html (2020, Apr 15)

[48] Beer, T. Despite 400,000 Fatalities, Trump Downplayed The Deadliness Of Covid Through His Final Days In Office. *Forbes.* https://www.forbes.com/sites/tommybeer/2021/01/20/despite-400000-fatalities-trump-downplayed-the-deadliness-of-covid-through-his-final-days-in-office/?sh=405669631764 (2021, Jan 20)

[49] Bursztyn L., Rao A., Roth C. & Yanagizawa-Drott D. Misinformation during a pandemic. University of Chicago, Becker Friedman Institute for Economics Working Paper No. 2020–44 https://bfi.uchicago.edu/wp-content/uploads/BFI_WP_202044.pdf (2020).

[50] Lippold JV, Laske JI, Hogeterp SA, Duke É, Grünhage T, Reuter M (2020). the role of personality, political attitudes and socio-demographic characteristics in explaining individual differences in fear of coronavirus: a comparison over time and across countries. Front. Psychol..

[51] Prince-Gibson, E. (2021, Mar 26). Did Israel’s Security State Fail the COVID Test? *Foreign Policy*. https://foreignpolicy.com/2021/03/26/israel-netanyahu-covid-haredim-security-fail/

[52] BBC. (2020, Sept 30). Coronavirus: Israel passes law to ban mass protests during lockdown. BBC. https://www.bbc.com/news/world-middle-east-54354826

[53] SAS Institute INC (2013). *SAS® 9.4 Statements: Reference.* Cary, NC: SAS Institute Inc.

[54] Coppock A, McClellan OA (2019). Validating the demographic, political, psychological, and experimental results obtained from a new source of online survey respondents. Res. Polit..

[55] Hayes AF (2017). Methodology in the social sciences. Introduction to mediation, moderation, and conditional process analysis: a regression-based approach.

[56] Galasso V, Pons V, Profeta P, Becher M, Brouard S, Foucault M (2020). Gender differences in COVID-19 attitudes and behavior: panel evidence from eight countries. Proc. Natl. Acad. Sci. U.S.A..

[57] Olcaysoy Okten, I., Gollwitzer, A., & Oettingen, G. . Gender differences in preventing the spread of coronavirus. Behavioral Science & Policy. Retrieved from https://behavioralpolicy.org/journal_issue/covid-19 (2020)

[58] Litman L, Robinson J, Abberbock T (2017). TurkPrime.com: a versatile crowdsourcing data acquisition platform for the behavioral sciences. Behav. Res. Methods.

[59] Chandler J, Rosenzweig C, Moss AJ, Robinson J, Litman L (2019). Online panels in social science research: expanding sampling methods beyond Mechanical Turk. Behav. Res. Methods.

[60] Cohen J (1988). Statistical power analysis for the behavioral sciences.

[61] Taylor S, Asmundson G (2021). Negative attitudes about facemasks during the COVID-19 pandemic: the dual importance of perceived ineffectiveness and psychological reactance. PLoS ONE.

[62] Bridgman A, Merkley E, Zhilin O, John L, Owen T, Ruths D (2021). Infodemic pathways: evaluating the role that traditional and social media play in cross-national information transfer. Front. Polit. Sci..

[63] Mietzner M (2020). Populist anti-scientism, religious polarisation, and institutionalised corruption: How Indonesia’s democratic decline shaped its COVID-19 response. J. Curr. Southeast Asian Affairs.

[64] Malka A, Soto CJ, Inzlicht M, Lelkes Y (2014). Do needs for security and certainty predict cultural and economic conservatism? A cross-national analysis. J. Personal. Soc. Psychol..

[65] Fawcett E (2020). Conservatism: the fight for a tradition.

[66] Ziblatt D (2017). Conservative political parties and the birth of modern democracy in Europe.

[67] Noël A, Thérien JP (2008). Left and right in global politics.

[68] Waytz A, Iyer R, Young L, Haidt J, Graham J (2019). Ideological differences in the expanse of the moral circle. Nat. Commun..

[69] Pickup M, Stecula D, van der Linden C (2020). Novel coronavirus, old partisanship: COVID-19 attitudes and behaviours in the United States and Canada. Can. J. Polit. Sci..

[70] Haug N, Geyrhofer L, Londei A (2020). Ranking the effectiveness of worldwide COVID-19 government interventions. Nat. Hum. Behav..

[71] Alfano V, Ercolano S (2020). The efficacy of lockdown against COVID-19: a cross-country panel analysis. Appl. Health Econ. Health Policy.

[72] Brauner JM, Mindermann S, Sharma M, Johnston D, Salvatier J, Gavenciak T, Stephenson AR, Leech G, Altman G, Mikulik V, Norman AJ, Monrad JT, Besiroglu T, Ge H, Hartwick MA, Teh YW, Chindelevitch L, Gal Y, Kulveit J (2021). Inferring the effectiveness of government interventions against COVID-19. Science.

[73] Bullock JG, Green DP, Ha SE (2010). Yes, but what's the mechanism? (don't expect an easy answer). J. Personal. Soc. Psychol..

[74] Sidanius J, Pratto F (1999). Social dominance: an intergroup theory of social hierarchy and oppression.

[75] Ho AK, Sidanius J, Kteily N, Sheehy-Skeffington J, Pratto F, Henkel KE, Foels R, Stewart AL (2015). The nature of social dominance orientation: theorizing and measuring preferences for intergroup inequality using the new SDO_7_ scale. J. Personal. Soc. Psychol..

[76] Clarke E, Klas A, Dyos E (2021). The role of ideological attitudes in responses to COVID-19 threat and government restrictions in Australia. Person. Individ. Differ..

[77] Prichard EC, Christman SD (2020). Authoritarianism, Conspiracy Beliefs, Gender and COVID-19: Links Between Individual Differences and Concern About COVID-19, Mask Wearing Behaviors, and the Tendency to Blame China for the Virus. Front. Psychol..

[78] Stenner K (2009). Three kinds of "conservatism.". Psychol. Inq..

