# Supplementary material for: Trust in scientific information mediates associations between conservatism and coronavirus responses in the U.S., but few other nations
Source: Sci Rep. 2022 Mar 8;12:3724. doi: 10.1038/s41598-022-07508-6 (PMC8904544; doi:10.1038/s41598-022-07508-6)
Supplement: Supplementary file 1 — Supplementary Information. [file 41598_2022_7508_MOESM1_ESM.pdf]

Trust in Scientific Information Mediates Associations between Conservatism and Coronavirus Responses in the U.S., but Few Other Nations

McLamore, Quinnehtukqut[1] & Syropoulos, Stylianos[1] (joint first-authorship), Leidner, Bernhard[1], Hirschberger, Gilad[2], Young, Kevin[1], Zein, Rizqy Amelia[3], Baumert, Anna[4,25], Bilewicz, Michal[5], Bilgen, Arda[20], van Bezouw, Maarten J.[6], Chatard, Armand[7], Chekroun, Peggy[8], Chinchilla, Juana[10], Choi, Hoon-Seok[9], Euh, Hyun[23], Gomez, Angel[10], Kardos, Peter[11], Khoo, Ying Hooi[12], Li, Mengyao[4], Légal, Jean-Baptiste[8], Loughnan, Steve[13], Mari, Silvia[14], Tan-Mansukhani, Roseann[15], Muldoon, Orla[16], Noor, Masi[17], Paladino, Maria Paola[24], Petrović, Nebojša[18], Selvanathan, Hema Preya[19], Uluğ, Özden Melis[20], Wohl, Michael J.[21], Yeung, Wai Lan Victoria[22], Burrows, B.[1]

[1]University of Massachusetts, Amherst, USA

[2]IDC, Herzliya, Israel

[3]Universitas Airlangga, Indonesia

[4] Max Planck Institute for Research on Collective Goods, Germany

[5]University of Warsaw, Poland

[6]Universiteit van Amsterdam, the Netherlands

[7]Université de Poitiers, France

[8]University of Paris Nanterre, France

[9] Sungkyunkwan University, Republic of Korea

[10] Universidad Nacional de Educacion a Distancia, Spain

[11]Bloomfield College, USA

[12]University of Malaya, Malaysia

[13]University of Edinburgh, UK

[14]University of Milano-Bicocca, Italy

[15]De La Salle University, Philippines

[16]University of Limerick, Republic of Ireland

[17]Keele University, UK

[18]University of Belgrade, Serbia

[19]University of Queensland, Australia

[20]University of Sussex, UK

[21]Carleton University, Canada

[22]Lingnan University Hong Kong

[23] University of Illinois at Urbana-Champaign, USA

[24]University of Trento, Italy

[25]University of Wuppertal, Germany

Corresponding Author:

Quinnehtukqut McLamore
University of Massachusetts Amherst, Psychological & Brain Sciences
Tobin Hall, 135 Hicks Way
Amherst, MA 01003
email: qmclamore@umass.edu

Supplementary Materials

Table S1.

Additional demographic information for Studies 1 and 2.

	Income				Education			
Study	*M*	*SD*	range	Descriptive Average	*M*	*SD*	range	Descriptive Average
1a	10.36	4.21	1-18	$50,000 - $59,999	3.40	1.06	1-5	Some College Education
1b	10.39	4.18	1-18	$50,000 - $59,999	3.41	1.05	1-5	Some College Education
2a	3.77	1.89	1-7	$50,000 - $70,000	3.87	1.59	1-8	2-year College Degree
2b	4.02	1.89	1-7	$50,000 - $70,000	3.99	1.64	1-8	2-year College Degree

Table S2

Demographic information and descriptive statistics for key variables for each country in Study 3

Country	Gender		Age	Support for Curtailments		COVID-19 Compliance		Trust in Science		Political Ideology	COVID-19 Concern
	Male	Female	M (SD)	M (SD)	α	M (SD)	α	M (SD)	α	M (SD)	M (SD)
United States	1787	1754	47.35 (17.56)	5.90 (1.65)	0.81	7.51 (1.55)	0.82	7.12 (1.63)	0.79	3.97 (1.82)	2.45 (1.11)
United Kingdom	449	450	48.10 (17.36)	6.37 (1.55)	0.83	7.81 (1.33)	0.77	6.21 (1.43)	0.78	3.88 (1.58)	2.37 (1.04)
Turkey	446	448	36.49 (11.82)	6.78 (1.60)	0.79	8.03 (1.20)	0.74	7.34 (1.69)	0.75	3.83 (1.54)	2.86 (1.07)
Serbia	570	619	43.91 (12.63)	5.29 (2.11)	0.85	7.22 (1.72)	0.73	6.27 (2.18)	0.76	3.35 (1.30)	1.87 (0.94)
Poland	434	452	47.09 (16.29)	5.84 (2.03)	0.89	7.18 (1.76)	0.83	6.90 (1.69)	0.79	3.59 (1.40)	2.58 (1.10)
Philippines	432	451	36.66 (14.07)	7.57 (1.40)	0.79	8.27 (1.09)	0.61	7.94 (1.34)	0.71	3.79 (1.40)	3.16 (1.05)
Netherlands	476	420	49.95 (17.65)	5.54 (1.75)	0.85	6.89 (1.14)	0.69	6.99 (1.58)	0.79	3.51 (1.30)	2.17 (1.02)
Malaysia	457	439	39.63 (13.44)	7.61 (1.17)	0.80	7.61 (1.28)	0.68	7.51 (1.34)	0.71	3.66 (1.39)	2.93 (1.04)
South Korea	482	428	46.44 (15.02)	5.84 (1.47)	0.81	7.20 (1.32)	0.80	7.09 (1.28)	0.74	3.83 (1.28)	2.43 (1.00)
Italy	436	454	48.64 (15.80)	5.93 (1.80)	0.84	7.51 (1.46)	0.77	6.87 (1.62)	0.71	3.83 (1.67)	2.53 (1.05)
Israel	445	555	38.46 (15.14)	6.17 (1.74)	0.82	6.99 (1.62)	0.76	6.89 (1.62)	0.71	4.73 (1.53)	2.44 (1.10)
Ireland	443	447	45.79 (15.93)	6.36 (1.68)	0.83	7.86 (1.31)	0.77	7.40 (1.53)	0.79	3.72 (1.24)	2.40 (1.04)
Indonesia	467	415	38.81 (13.31)	7.34 (1.28)	0.76	7.84 (1.27)	0.80	7.52 (1.33)	0.67	2.83 (1.36)	3.22 (0.95)
Hungary	424	466	40.05 (16.11)	5.41 (1.92)	0.85	7.04 (1.27)	0.76	6.76 (1.75)	0.72	3.95 (1.50)	2.15 (1.03)
Germany	449	448	47.04 (17.22)	5.24 (1.87)	0.84	7.19 (1.61)	0.76	6.86 (1.77)	0.82	3.71 (1.10)	2.14 (1.06)
France	448	441	49.47 (16.24)	6.02 (1.60)	0.78	7.44 (1.46)	0.74	6.85 (1.55)	0.68	4.20 (1.81)	2.59 (1.12)
Spain	459	442	47.27 (15.04)	6.29 (1.65)	0.81	7.73 (1.34)	0.76	7.31 (1.58)	0.75	3.59 (1.60)	2.49 (1.05)
Canada	438	456	46.86 (17.00)	6.33 (1.60)	0.83	7.67 (1.41)	0.80	7.28 (1.48)	0.80	3.50 (1.71)	2.32 (1.04)
Australia	422	449	46.80 (17.54)	6.60 (1.54)	0.84	7.43 (1.48)	0.80	7.33 (1.43)	0.82	4.19 (1.23)	2.29 (1.06)

Table S3.

Bivariate correlations between conservative/right wing ideology and other measures in each country for wave 1 of Study 3.

Country	N	COVID-19 Concern	Trust in Science	COVID-19 Compliance	Civil Curtailments
United States	1167	**-0.15***	**-0.17***	**-0.16***	**-0.16***
Canada	291	**-0.14***	**-0.30***	**-0.18****	**-0.22***
United Kingdom	337	**-0.14***	-0.01	0.07	0.06
Turkey	294	**-0.14***	-0.03	0.08	0.08
Serbia	599	0.03	-0.02	-0.04	0.08
Poland	294	0.09	**-0.20****	-0.02	0.09
Philippines	298	0.02	-0.04	**0.21***	**0.13***
Netherlands	297	0.06	0.06	0.03	0.12
Malaysia	289	0.01	0.08	0.06	0.00
South Korea	298	0.02	0.00	-0.02	-0.03
Italy	297	0.12	-0.09	0.10	**0.15***
Israel	358	**0.13***	0.08	0.10	**0.41***
Ireland	293	0.02	-0.01	-0.03	0.02
Indonesia	279	0.00	**-0.15***	**-0.15***	-0.04
Hungary	294	-0.03	-0.04	0.02	**0.19****
Germany	302	0.01	**-0.12***	**-0.13***	0.11
France	294	0.11	-0.06	-0.13	0.11
Spain	298	-0.02	-0.07	0.06	0.03
Australia	295	-0.03	0.04	-0.01	-0.01

Note: * $p < .05$, ** $p < .01$, *** $p < .001$. Bolded values depicting significant correlations.

Table S4.

Bivariate correlations between conservative/right wing ideology and other measures in each country for wave 2 of Study 3.

Country	N	COVID-19 Concern	Trust in Science	COVID-19 Compliance	Civil Curtailments
United States	1180	**-0.09*** **	**-0.21*** **	**-0.09** **	**-0.07***
Canada	311	**-0.16***	**-0.34*** **	**-0.24*** **	-0.11
United Kingdom	297	-0.05	0.00	0.10	**0.13***
Turkey	298	-0.08	0.11	-0.03	0.03
Serbia	286	-0.02	-0.08	-0.07	0.09
Poland	294	0.02	-0.06	0.02	**0.16** **
Philippines	294	0.01	-0.07	0.03	-0.03
Netherlands	296	0.10	-0.09	-0.08	0.08
Malaysia	297	-0.02	-0.08	-0.05	0.00
South Korea	312	-0.06	**0.14***	-0.02	-0.08
Italy	292	0.01	-0.10	0.00	0.03
Israel	346	0.08	**-0.13***	0.02	**0.21*** **
Ireland	295	-0.02	-0.08	**-0.15***	-0.03
Indonesia	293	**-0.22*** **	**-0.22*** **	**-0.18** **	-0.05
Hungary	297	-0.04	0.10	-0.06	**0.17** **
Germany	297	0.04	**-0.25*** **	**-0.15***	0.09
France	300	0.07	-0.02	-0.04	0.13
Spain	296	-0.03	0.05	0.02	0.04
Australia	314	**0.17***	0.03	0.05	**0.30*** **

Note: * p < .05, ** p < .01, *** p < .001. Bolded values depicting significant correlations.

Table S5.

Bivariate correlations between conservative/right wing ideology and other measures in each country for wave 3 of Study 3.

Country	N	COVID-19 Concern	Trust in Science	COVID-19 Compliance	Civil Curtailments
United States	1236	**-0.27*****	**-0.29*****	**-0.15*****	**-0.16*****
Canada	310	-0.08	**-0.20*****	0.02	-0.08
United Kingdom	312	-0.02	0.04	0.08	**0.14***
Turkey	322	0.00	0.08	0.00	0.10
Serbia	320	-0.09	-0.10	**-0.16****	-0.03
Poland	310	0.02	-0.04	0.02	**0.15****
Philippines	355	-0.02	-0.08	-0.02	0.07
Netherlands	326	**0.17****	0.03	0.10	**0.12***
Malaysia	338	**-0.13***	-0.02	0.00	0.04
South Korea	320	0.10	-0.04	-0.05	**-0.13***
Italy	309	-0.01	**-0.17****	0.03	**0.18****
Israel	381	0.02	-0.02	-0.03	**0.27*****
Ireland	329	-0.10	-0.12	-0.08	0.01
Indonesia	356	-0.05	0.08	-0.10	0.01
Hungary	320	-0.03	0.11	-0.04	**0.19****
Germany	308	0.01	0.07	-0.05	0.01
France	314	**-0.16***	**-0.17***	-0.05	0.11
Spain	332	-0.06	**-0.25*****	-0.02	-0.06
Australia	313	0.01	-0.12	0.04	0.09

Note: * $p < .05$, ** $p < .01$, *** $p < .001$. Bolded values depicting significant correlations.

Table S6.

Bivariate correlations between conservative/right wing ideology and other measures in each country across all waves of Study 3.

Country	N	COVID-19 Concern	Trust in Science	COVID-19 Compliance	Civil Curtailments
United States	3583	**-0.16*****	**-0.22*****	**-0.13*****	**-0.13*****
Canada	912	**-0.10****	**-0.28*****	**-0.14*****	**-0.13*****
United Kingdom	946	-0.07	0.01	**0.08***	**0.11****
Turkey	914	-0.08	0.05	0.02	**0.08***
Serbia	1205	-0.01	**-0.06***	**-0.08***	0.05
Poland	898	0.04	**-0.10****	0.02	**0.14*****
Philippines	947	0.00	-0.06	**0.08***	0.06
Netherlands	919	**0.09***	0.00	0.02	**0.11****
Malaysia	924	-0.05	-0.02	0.00	0.01
South Korea	930	-0.01	0.03	-0.03	**-0.08***
Italy	898	0.02	**-0.12****	0.06	**0.13*****
Israel	1086	**0.08***	-0.02	0.02	**0.29*****
Ireland	917	-0.01	-0.07	**-0.10****	0.00
Indonesia	928	**-0.10****	**-0.16*****	**-0.14*****	-0.01
Hungary	911	-0.02	0.06	-0.03	**0.18*****
Germany	907	0.02	**-0.10****	**-0.10****	**0.08***
France	908	0.02	**-0.08***	-0.08	**0.11****
Spain	926	-0.04	**-0.09****	0.02	0.00
Australia	922	0.04	-0.02	0.03	**0.15*****

Note: * $p < .05$, ** $p < .01$, *** $p < .001$. Bolded values depicting significant correlations.

Table S7.

Meta-correlations between conservative/right wing ideology and concerns about COVID-19 in each country across all waves of Study 3.

	Mean *r*	*SE*	*Z*	Lower 95% CI	Upper 95% CI
United States	**-0.17*****	0.02	-10.43	-0.20	-0.14
Canada	**-0.13*****	0.03	-3.82	-0.19	-0.06
United Kingdom	**-0.07***	0.03	-2.22	-0.14	-0.01
Turkey	**-0.07***	0.03	-2.15	-0.14	-0.01
Serbia	-0.01	0.03	-0.47	-0.07	0.04
Poland	0.04	0.03	1.28	-0.02	0.11
Philippines	0.00	0.03	0.06	-0.06	0.07
Netherlands	**0.11*****	0.03	3.40	0.05	0.18
Malaysia	-0.05	0.03	-1.55	-0.12	0.01
South Korea	0.02	0.03	0.63	-0.04	0.09
Italy	0.04	0.03	1.18	-0.03	0.11
Israel	**0.08***	0.03	2.48	0.02	0.13
Ireland	-0.04	0.03	-1.09	-0.10	0.03
Indonesia	**-0.09****	0.03	-2.72	-0.15	-0.03
Hungary	-0.03	0.03	-1.00	-0.10	0.03
Germany	0.02	0.03	0.59	-0.05	0.09
France	0.00	0.03	0.09	-0.06	0.07
Spain	-0.04	0.03	-1.14	-0.10	0.03
Australia	0.05	0.03	1.58	-0.01	0.12

Note: * $p < .05$, ** $p < .01$, *** $p < .001$. Bolded values depicting significant correlations.

Table S8.

Meta-correlations between conservative/right wing ideology and trust in science in each country across all waves of Study 3.

	Mean *r*	*SE*	*Z*	Lower 95% CI	Upper 95% CI
United States	**-0.23*** **	0.02	-13.71	-0.26	-0.20
Canada	**-0.28*** **	0.03	-8.70	-0.36	-0.22
United Kingdom	0.01	0.03	0.39	-0.05	0.08
Turkey	0.05	0.03	1.65	-0.01	0.12
Serbia	-0.06	0.03	-1.93	-0.11	0.00
Poland	**-0.10***	0.03	-2.99	-0.17	-0.03
Philippines	-0.06	0.03	-1.98	-0.13	0.00
Netherlands	0.00	0.03	0.03	-0.06	0.07
Malaysia	-0.01	0.03	-0.24	-0.07	0.06
South Korea	0.04	0.03	1.11	-0.03	0.10
Italy	**-0.12*** **	0.03	-3.62	-0.19	-0.06
Israel	-0.02	0.03	-0.73	-0.08	0.04
Ireland	**-0.07***	0.03	-2.04	-0.14	0.00
Indonesia	**-0.08***	0.03	-2.58	-0.15	-0.02
Hungary	0.06	0.03	1.76	-0.01	0.12
Germany	**-0.10** **	0.03	-3.00	-0.17	-0.03
France	**-0.09***	0.03	-2.56	-0.15	-0.02
Spain	**-0.10** **	0.03	-2.97	-0.16	-0.03
Australia	-0.02	0.03	-0.54	-0.08	0.05

Note: * $p < .05$, ** $p < .01$, *** $p < .001$. Bolded values depicting significant correlations.

Table S9.

Meta-correlations between conservative/right wing ideology and compliance with COVID-19 recommendations in each country across all waves of Study 3.

	Mean *r*	*SE*	*Z*	Lower 95% CI	Upper 95% CI
United States	**-0.14*** **	0.02	-8.25	-0.17	-0.11
Canada	**-0.13*** **	0.03	-4.06	-0.20	-0.07
United Kingdom	**0.08* **	0.03	2.55	0.02	0.15
Turkey	0.02	0.03	0.59	-0.05	0.09
Serbia	**-0.08* **	0.03	-2.75	-0.14	-0.02
Poland	0.01	0.03	0.21	-0.06	0.07
Philippines	**0.07* **	0.03	2.12	0.01	0.13
Netherlands	0.02	0.03	0.59	-0.05	0.08
Malaysia	0.00	0.03	0.08	-0.06	0.07
South Korea	-0.03	0.03	-0.92	-0.09	0.03
Italy	0.05	0.03	1.39	-0.02	0.11
Israel	0.03	0.03	0.95	-0.03	0.09
Ireland	**-0.08* **	0.03	-2.29	-0.15	-0.01
Indonesia	**-0.14*** **	0.03	-4.29	-0.21	-0.08
Hungary	-0.03	0.03	-0.82	-0.09	0.04
Germany	**-0.11*** **	0.03	-3.30	-0.18	-0.04
France	**-0.07* **	0.03	-2.18	-0.14	-0.01
Spain	0.02	0.03	0.56	-0.05	0.08
Australia	0.03	0.03	0.83	-0.04	0.09

Note: * $p < .05$, ** $p < .01$, *** $p < .001$. Bolded values depicting significant correlations.

Table S10.

Meta-correlations between conservative/right wing ideology and compliance with support for civil curtailments to prevent the spread of COVID-19 in each country across all waves of Study 3.

	Mean *r*	*SE*	*Z*	Lower 95% CI	Upper 95% CI
United States	**-0.13***	0.02	-7.86	-0.16	-0.10
Canada	**-0.14***	0.03	-4.11	-0.20	-0.07
United Kingdom	**0.11***	0.03	3.34	0.05	0.17
Turkey	**0.07***	0.03	2.14	0.01	0.14
Serbia	0.05	0.03	1.85	0.00	0.11
Poland	**0.13***	0.03	4.03	0.07	0.20
Philippines	0.06	0.03	1.79	-0.01	0.12
Netherlands	**0.11***	0.03	3.24	0.04	0.17
Malaysia	0.02	0.03	0.63	-0.04	0.09
South Korea	**-0.08***	0.03	-2.47	-0.15	-0.02
Italy	0.12	0.03	3.65	0.06	0.19
Israel	**0.30***	0.03	10.13	0.25	0.37
Ireland	0.00	0.03	0.10	-0.06	0.07
Indonesia	-0.02	0.03	-0.73	-0.09	0.04
Hungary	**0.18***	0.03	5.57	0.12	0.25
Germany	**0.07***	0.03	2.09	0.00	0.14
France	**0.12***	0.03	3.51	0.05	0.18
Spain	0.00	0.03	0.03	-0.06	0.07
Australia	**0.13***	0.03	4.01	0.07	0.20

Note: * $p < .05$, ** $p < .01$, *** $p < .001$. Bolded values depicting significant correlations.

Table S11.

Bivariate correlations between trust in science and each COVID-19 variable included in Study 3.

Trust in Science for	Wave 1				Wave 2				Wave 3				Across Waves			
	N	Con.	Comp.	Curt.	N	Con.	Comp.	Curt.	N	Con.	Comp.	Curt.	N	Con.	Comp.	Curt.
United States	1167	.18***	.48***	.42***	1180	.11**	.40***	.32***	1236	.31***	.43***	.35***	3583	.19***	.44***	.36***
Canada	291	.05	.54***	.44***	311	.02	.41***	.36***	310	.26***	.35***	.34***	912	.10**	.43***	.38***
United Kingdom	337	.17**	.28***	.24***	297	.23***	.35***	.21***	312	.17**	.43***	.29***	946	.16***	.39***	.26***
Turkey	294	.09	.41***	.29***	298	.15**	.21***	.14*	322	.11	.34***	.24***	914	.14***	.28***	.21***
Serbia	599	.08	.27***	.36***	286	-.01	.39***	.37***	320	.20***	.34***	.33***	1205	.03	.34***	.37***
Poland	294	.05	.44***	.22***	294	.24***	.48***	.38***	310	.31***	.40***	.32***	898	.19***	.44***	.31***
Philippines	298	.09	.33***	.36***	294	.13	.34***	.39***	355	.14*	.24***	.18***	947	.12***	.29***	.31***
Netherlands	297	.14*	.39***	.30***	296	.10	.44***	.26***	326	.14*	.23***	.10	919	.09**	.35***	.22***
Malaysia	289	.12	.40***	.52***	297	.13*	.38***	.41***	338	.05	.39***	.38***	924	.13***	.38***	.44***
South Korea	298	.21***	.38***	.19***	312	.11	.33***	.07	320	.09	.44***	.19***	930	.12***	.38***	.15***
Italy	297	.21***	.36***	.41***	292	.13*	.34***	.26***	309	.15*	.28***	.21***	898	.14***	.33***	.29***
Israel	358	.03	.23***	.36***	346	.12*	.19**	.15**	381	.12*	.23***	.24***	1086	.07*	.22***	.26***
Ireland	293	.06	.46***	.39***	295	.04	.45***	.29***	329	.24***	.45***	.35***	917	.11**	.45***	.34***
Indonesia	279	.00	.36***	.20***	293	.24***	.30***	.35***	356	.12*	.27***	.24***	928	.12***	.31***	.27***
Hungary	294	.13*	.29***	.15***	297	-.11	.31***	.25***	320	.19**	.29***	.20***	911	.08*	.28***	.20***
Germany	302	.02	.28***	.27***	297	.09	.53***	.27***	308	.23***	.42***	.22***	907	.10*	.40***	.25***
France	294	.03	.35***	.30***	300	.01	.36***	.22***	314	-.07	.22***	-.05	908	-.02	.31***	.16***
Spain	298	.02	.41***	.32***	296	.14*	.35***	.27***	332	.10	.28***	.28***	926	.08*	.33***	.28***
Australia	295	-.01	.53***	.40***	314	.07	.44***	.32***	313	.11	.44***	.42***	922	.06	.46***	.38***

Note: * $p < .05$, ** $p < .01$, *** $p < .001$. Con = Concerns for contracting COVID-19. Comp = Compliance to COVID-19 recommendations. Curt = Support for civil curtailments to prevent the spread of COVID-19.

Figures S1-S3.

Visual depiction of the meta-correlations across the three waves for each country between trust in science and concerns about contracting the coronavirus (first graph), compliance with COVID-19 regulations (second graph), and support for civil curtailments (third graph). Bolded values depict significant associations.

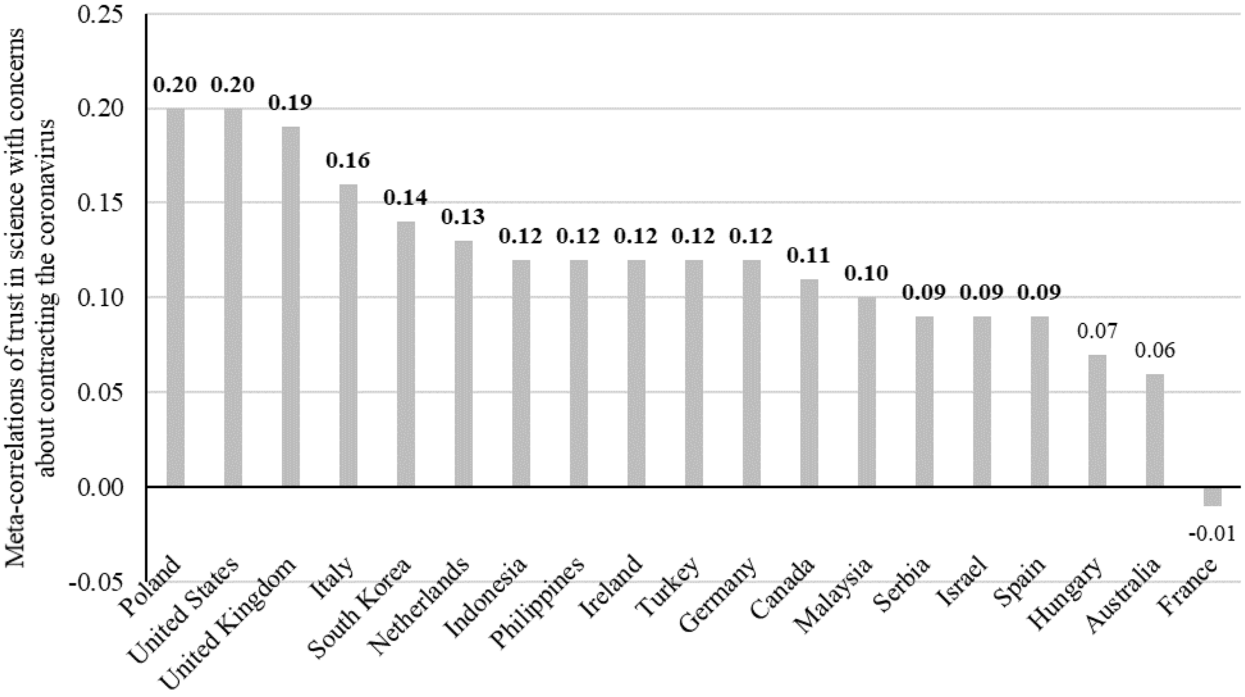

Figure S1. Meta-correlations of trust in science with concerns about contracting the coronavirus in each country samples in Study 3. Bolded coefficients are significant (p <.05).

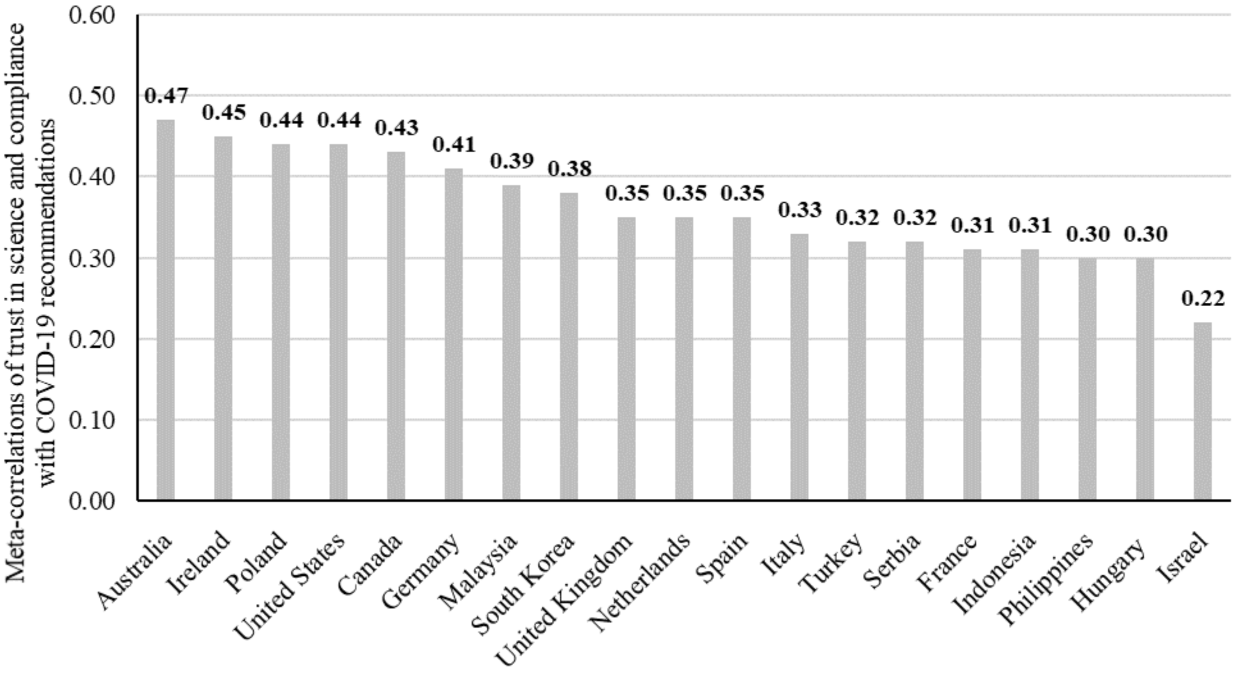

Figure S2. Meta-correlations of trust in science and compliance with coronavirus recommendations in Study 3. Bolded coefficients are significant (*p* <.05).

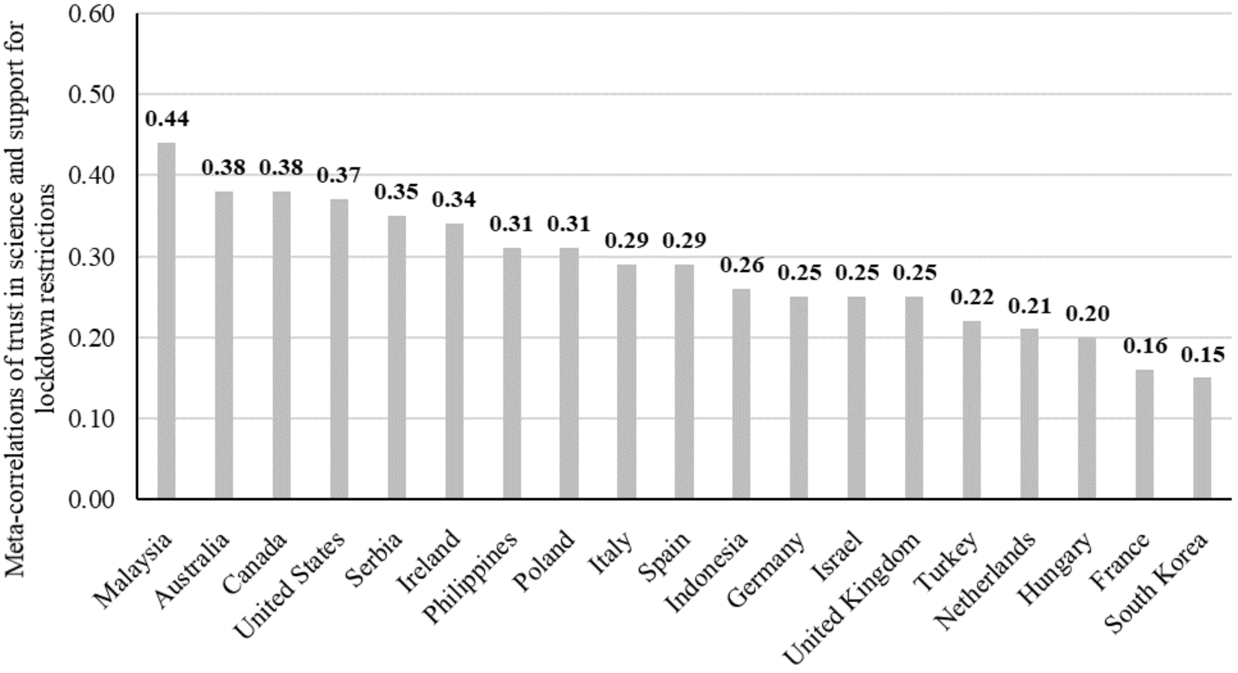

Figure S3. Meta-correlations of trust in science with support for lockdown restrictions in each country samples in Study 3. Bolded coefficients are significant (p <.05).
